# Comparative Analysis of Korean Human Gut Microbiota by Barcoded Pyrosequencing

**DOI:** 10.1371/journal.pone.0022109

**Published:** 2011-07-29

**Authors:** Young-Do Nam, Mi-Ja Jung, Seong Woon Roh, Min-Soo Kim, Jin-Woo Bae

**Affiliations:** 1 Department of Life and Nanopharmaceutical Sciences and Department of Biology, Kyung Hee University, Dongdaemun-gu, Seoul, Republic of Korea; 2 Traditional Food Research Group, Korea Food Research Institute, Sungnam, Republic of Korea; AC Camargo Cancer Hospital, Brazil

## Abstract

Human gut microbiota plays important roles in harvesting energy from the diet, stimulating the proliferation of the intestinal epithelium, developing the immune system, and regulating fat storage in the host. Characterization of gut microbiota, however, has been limited to western people and is not sufficiently extensive to fully describe microbial communities. In this study, we investigated the overall composition of the gut microbiota and its host specificity and temporal stability in 20 Koreans using 454-pyrosequencing with barcoded primers targeting the V1 to V3 region of the bacterial 16S rRNA gene. A total of 303,402 high quality reads covered each sample and 8,427 reads were analyzed on average. The results were compared with those of individuals from the USA, China and Japan. In general, microbial communities were dominated by five previously identified phyla: Actinobacteria, Firmicutes, Bacteroidetes, Fusobacteria, and Proteobacteria. UPGMA cluster analysis showed that the species composition of gut microbiota was host-specific and stable over the duration of the test period, but the relative abundance of each member fluctuated. 43 core Korean gut microbiota were identified by comparison of sequences from each individual, of which 15 species level phylotypes were related to previously-reported butyrate-producing bacteria. UniFrac analysis revealed that human gut microbiota differed between countries: Korea, USA, Japan and China, but tended to vary less between individual Koreans, suggesting that gut microbial composition is related to internal and external characteristics of each country member such as host genetics and diet styles.

## Introduction

After the completion of the Human Genome Project (HGP), many scientists were disappointed by the announcement that the human genome contained only around 20,000 protein coding genes rather than the 100,000 originally estimated because the number of gene was thought to be insufficient to solve the mysteries of human health and disease. Therefore, after the HGP, Human Microbiome Project (HMP) was initiated to fill a gap between our current understanding derived from HGP and actual physiological phenomenon not regulated by human but microbes and HMP created a new view of ourselves as ‘super-organisms’ consisting of a human host and thousands of microbial symbionts [1].

All surfaces of the human body, including the skin, mucosal surface and genital and gastrointestinal tracts are occupied by habitat-specific microorganisms whose cells outnumber those of the human host by ten times [2]. The colon contains between 10^11^ and 10^12^ microbial cells per ml and this extremely high population density is associated with a number of total genes that is two orders of magnitude higher than that contained in the human genome [3]. Moreover, recent study estimated 9,000,000 unique human gut microbial genes with culture-independent sequence data and complete human gut bacterial genomes [4]. Co-evolution with these great microbial ecosystems serves important functions for the human host by presenting nutrients from diets [5], resisting the colonization of pathogens [6], stimulating the proliferation of the intestinal epithelium [7], and regulating fat storage [8]. In addition, numerous diseases, including type 1 diabetes (T1D) [9], inflammatory bowel disease (IBD) [10], and gastric and colonic cancers [11], [12], have been shown to be linked to dysbiosis of microbial communities.

Due to the importance of gut microbiota for human health, these populations have been analyzed intensely using both culture-dependent and culture-independent methods. However, it is generally appreciated that fewer than one percent of microorganisms can be successfully cultivated from most environments [13] and only 20–40% of species have been cultured from the human intestine, despite it being one of the most well-studied communities [2]. However, culture-independent molecular techniques have been applied that greatly revolutionized our understanding of gut microbiota. In particular, these methods are based on the small subunit ribosomal RNA (16S rRNA) gene and include processes such as denaturing gel electrophoresis (DGGE) [14], fluorescent *in situ* hybridization [15], microarray analysis [16], and PCR cloning and sequencing [17]. Of the molecular methods, those sequencing the full-length of the 16S rDNA gene through PCR and cloning provide the most powerful taxonomic resolution [17], but studies using the conventional dideoxy Sanger sequencing method have been forced to use small sample sizes due to its high cost and labor input. Consequently, only dominant members of the microbial communities have been described using this approach, leaving the large and diverse but rarer biosphere largely untouched [18].

However, recent advances in sequencing technology, such as the 454 pyrosequencing approach, are changing the way in which microbial communities are studied. These new sequence-by-synthesis methods provide a faster and simpler way for microbial communities to be analyzed by enabling hundreds of thousands of nucleotide sequences to be examined at a fraction of the cost of Sanger sequencing methods [18], [19]. These new approaches have been successfully applied to characterize the microbial diversity in various regions of the human body, including the skin [20], oral cavity [21], vagina [22], and intestinal tract [23], [24], [25].

While large studies using deep sequencing methods have revealed a greater diversity of human microbiota than previously estimated by other molecular methods, even these studies have proved insufficient to fully understand these communities [23], [24], [25]. A principal reason for this is that each person possesses their own personalized fingerprint of gut flora [2], which includes hundreds of microbial species that are present in a combination unique to each individual [26], [27]. Human microbial communities are affected by external factors such as lifestyle [28], [29], dietary patterns [30], [31], antibiotic usage [32], [33], and host genotype [24], [34]. However, recent studies using deep sequencing analysis have tended to sample Europeans [21], [25] and people from the USA [24], [35]. In contrast, microbial communities in eastern people have not yet been investigated. This imbalance needs to be addressed to more fully understand the interactions between humans and their gut microbiota. We examined the composition and intra- and inter-individual variation in the fecal microbiota of Koreans in the present study using the high-throughput 454 pyrosequencing technique. These findings were compared with the characteristics of previously reported gut microbial communities of people from other countries.

## Results

### Comparison of phylotype coverage and diversity estimation between samples

After quality control processes filtered out reads containing incorrect primer or barcode sequences and sequences that were shorter than 250 nucleotides or with more than one ambiguous base, a total of 303,402 high quality sequences were obtained. Each individual sample was covered by an average of 8,427 reads (range = 2,147−19,942, SD = 3,878) (Table 1). The depth of resolution in this sequencing analysis was reduced by the removal of potential read artifacts (ambiguous base filtering), elimination of incorrectly barcoded reads, and the pooling of 36 samples on a single pyrosequencing plate. For the estimation of gut microbial diversity, all the pyrosequencing reads were subjected to OTU determination. When all sequences were clustered with representatives under conditions demanding 90% to 100% sequence identity, the number of operational taxonomic units (OTUs) varied between 2,284 and 62,095 (Figure S1). The number of OTUs from each individual at the various similarity cut-off levels averaged 2,157 (SD = 851; 100%), 771 (SD = 313; 97%), and 499 (SD = 212; 95%), respectively (Table S1). A 97% sequence identity of the 16S rRNA gene is commonly used to determine species level phylotypes [36]. Meanwhile, the 3% sequence variation seen within a short hyper-variable region of the small subunit (SSU) rRNA gene may differ from the variation apparent in the full length SSU rRNA gene. This may arise because the correlation between genetic dissimilarities can vary according to the location of the sequence on the gene and the kind of organisms [21]. Nevertheless, most studies investigating microbial diversity to date have used a 3% sequence dissimilarity cutoff value to judge species level phylotypes [21], [37], [38]. Therefore, in the rest of this report, a 3% genetic distance is used to define OTUs to retain consistency with other studies using deep sequencing methods.

**Table 1:** Number of sequences analyzed, observed diversity richness (OTUs), estimated OTU richness (ACE and Chao 1), diversity index (Shannon), and estimated sample coverage for 16S rRNA libraries of Korean fecal samples.

Sample ID	Read	Unique	OTUs^a^	Estimated OTU richness		Shannon^b^	ESC^c^
				Chao 1	Ace		
A0	8598	3037	1179	2310 (2074,2608)	2826 (2721,2939)	5.83	0.86
A1	12189	4073	1508	2503 (2317,2732)	3224 (3131,3322)	6.18	0.88
A2	8218	2654	1048	1719 (1576,1900)	2289 (2188,2399)	5.71	0.87
B0	15508	2421	652	1120 (993,1293)	1416 (1368,1467)	3.67	0.96
B1	9814	1834	466	847 (726,1026)	981 (892,1087)	3.57	0.95
B2	5214	1322	408	741 (633,900)	929 (837,1041)	4.08	0.92
C0	7741	1934	590	1136 (979,1357)	1287 (1250,1325)	4.61	0.92
C1	13568	3006	702	1099 (993,1244)	1331 (1287,1379)	4.61	0.95
C2	10039	1921	570	1062 (922,1258)	1304 (1268,1342)	3.94	0.94
D0	7196	1897	626	1051 (937,1207)	1392 (1349,1437)	4.58	0.91
D1	8112	2388	756	1295 (1157,1479)	1607 (1533,1687)	5.16	0.91
D2	8232	2394	741	1335 (1183,1540)	1779 (1689,1876)	5.06	0.91
E0	4963	1756	673	1257 (1105,1461)	1697 (1636,1762)	5.31	0.86
E1	13009	3563	1001	1599 (1464,1774)	2077 (1988,2174)	5.13	0.92
E2	10435	2900	829	1631 (1434,1893)	2032 (1952,2119)	4.79	0.92
F0	8482	1982	540	932 (815,1098)	1163 (1065,1280)	4.22	0.94
F1	6281	1509	488	886 (764,1061)	1103 (1002,1224)	4.04	0.92
F2	5518	1421	445	706 (622,830)	841 (767,931)	4.18	0.92
G0	5833	1763	468	775 (681,909)	1037 (940,1156)	4.56	0.92
G1	3935	1438	492	978 (829,1193)	1213 (1100,1348)	5.03	0.87
G2	5680	1955	624	1085 (961,1255)	1608 (1472,1766)	5.28	0.89
H0	3405	1078	338	540 (471,647)	553 (491,642)	4.29	0.90
H1	3706	1269	357	541 (477,640)	673 (608,754)	4.44	0.90
H2	13973	3610	943	1643 (1476,1861)	2071 (1980,2170)	5.28	0.93
I	7151	2603	948	1592 (1448,1779)	2102 (2012,2199)	5.37	0.87
J	10805	3735	1356	2396 (2189,2653)	2811 (2725,2902)	5.91	0.87
K	10841	3680	1280	2147 (1974,2364)	2709 (2609,2817)	5.89	0.88
L	4007	1720	778	1410 (1256,1613)	1797 (1700,1904)	5.76	0.81
M	9772	2893	1030	1784 (1615,2002)	2160 (2074,2254)	5.61	0.89
N	6404	2247	725	1199 (1076,1366)	1530 (1454,1613)	5.24	0.89
O	6295	1819	667	1038 (938,1175)	1294 (1240,1354)	5.03	0.89
P	2147	952	387	814 (673, 1024)	1088 (973, 1226)	5.10	0.82
Q	8067	2770	903	1518 (1373, 1709)	1822 (1742, 1910)	5.52	0.89
R	19942	5149	1358	2333 (2139, 2576)	2991 (2884, 3106)	5.63	0.93
S	4717	1767	684	1190 (1060, 1365)	1627 (1563, 1695)	5.48	0.85
T	13605	3876	1212	2210 (2001, 2473)	2772 (2658, 2895)	5.64	0.91

Abbreviations: ESC, estimated sample coverage; OTU, operational taxonomic unit.

a Calculated with MOTHUR at the 3% distance level.

b Shannon diversity index calculated with MOTHUR (3% distance).

c ESC: G = 1−n/N, where n is the number of phylotypes and N is the total number of sequences.

Values in brackets are 95% confidence intervals as calculated by MOTHUR.

**Figure 1:**
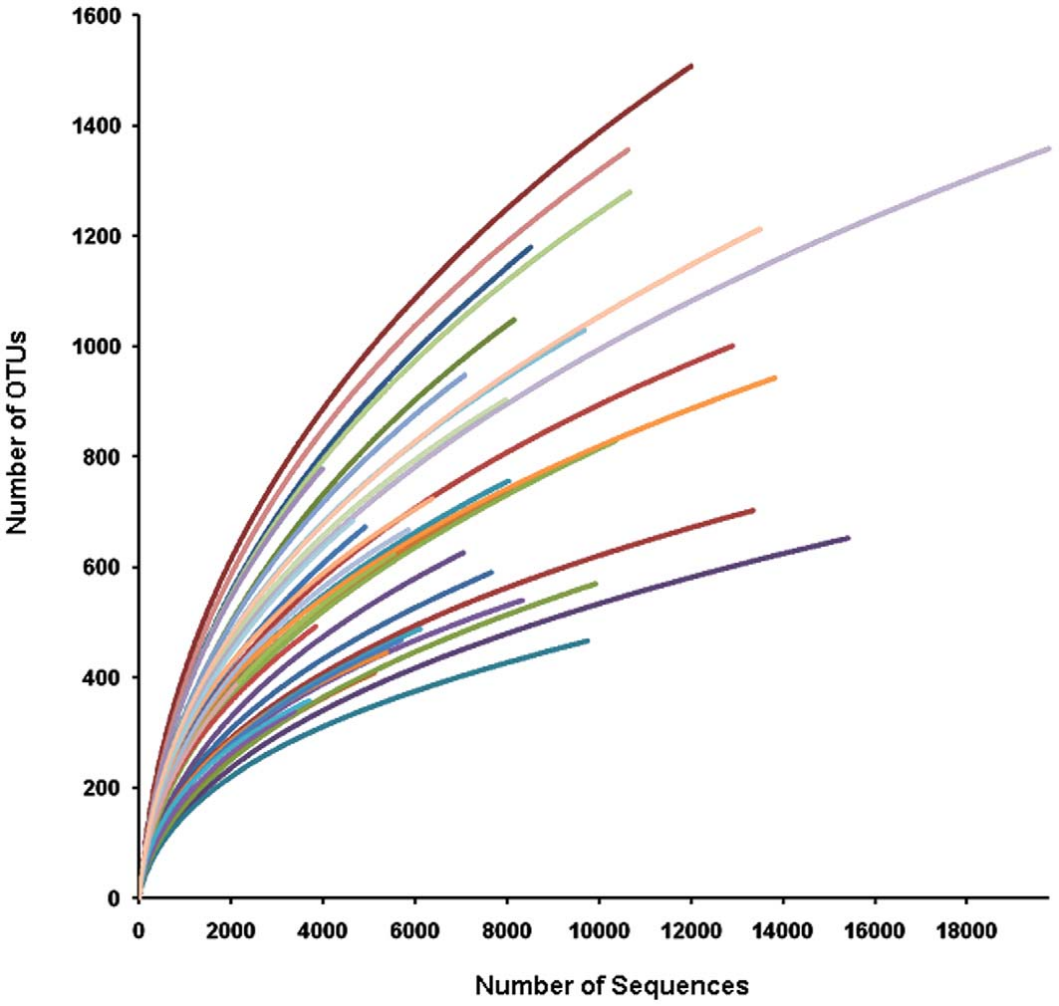
Rarefaction analysis of V1/V2 pyrosequencing reads of the 16S rRNA gene in fecal microbiota from 36 Korean samples. Rarefaction curves were constructed at a 97% sequence similarity cut-off value by MOTHUR.

When a rarefaction analysis was carried out to determine whether all the OTUs present in the datasets had been sufficiently recovered in the pyrosequencing study, individual rarefaction curves showed a similar pattern of reaching plateau but failing to reach a saturation phase (Fig. 1). This suggests that a large number of unseen OTUs still existed in the original samples and more sequencing effort may be required to detect any additional phylotypes. The number of OTUs in each sample estimated by ACE and Chao1 richness estimator was considerably higher than the number of observed OTUs (covering 39–64% and 34–52% of the estimated richness, respectively). OTU numbers generated by the Chao1 estimator suggested that there were between 184 and 1,131 additional phylotypes with a final richness of between 540 and 2,503 in each sample. As shown in Figure S2, the number of unique sequences in each sample generally rose in association with the sequence read size (R^2^ = 0.747) and the number of phylotypes also proportionately increased as the number of unique sequences rose (R^2^ = 0.842). Therefore, even for the samples with the largest set of sequences analyzed (individual R), further sequencing effort would probably detect at least 975 additional phylotypes. With the current sequencing effort, discordance between estimated and observed richness may be due to the effects of rare species. Microbial communities naturally contain a large number of rare species and a small number of abundant species [39], [40]. When the analysis of rank abundance for bacterial OTUs derived from all sequence reads was performed, Korean gut microbiota showed the greatest diversity of rare species (Figure S3). Good's coverage of each individual sample was used to estimate the completeness of sampling by a probability calculation based on a randomly selected amplicon sequence. The coverage ranged from 81 to 96% (average = 90%; SD = 4%) with a 97% species level phylotype threshold. For the overall sequence set, a 90% coverage value indicates that 10 additional phylotypes would be expected for every 100 additional sequencing efforts. However, the majority of bacterial phylotypes present in the Korean fecal samples were likely to have been identified in this study. The ecological diversity of Korean fecal microbiota was also estimated by the Shannon diversity index from the OTU data for each sample. The diversity index varied from 3.57 to 6.18 with an average of 4.99 (SD = 0.69). The rarefaction analysis of these estimates revealed that the diversity of each sample had reached a stable value (Figure S4).

### Korean gut microbial communities

**Figure 2:**
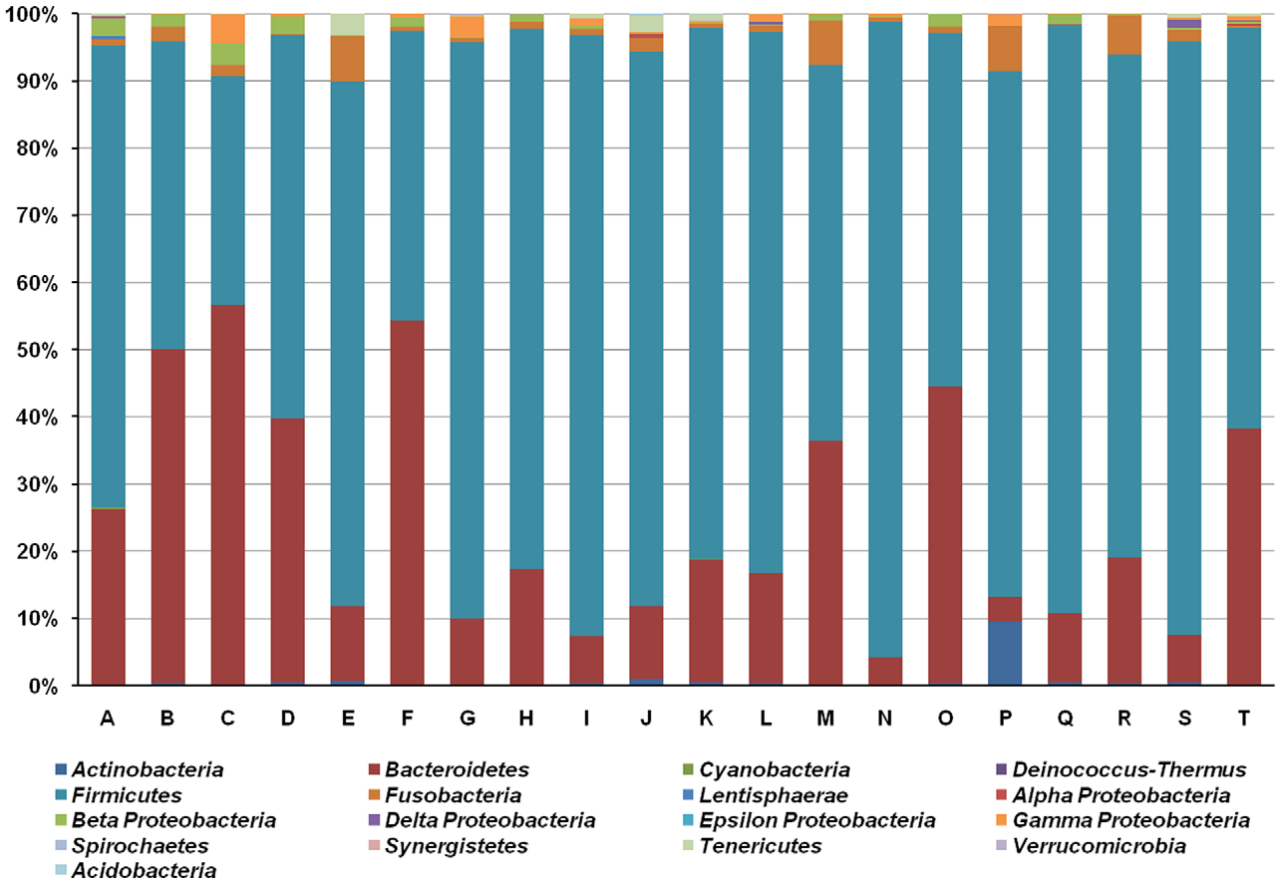
Relative abundance of partial sequences of bacterial 16S rRNA genes from fecal samples of 20 Koreans classified by MOTHUR at the phylum level with a modified 16S rRNA database from the Ribosomal Database Project (RDP).

Figure 2 shows the relative abundance of Korean gut microbiota at the phylum level. For the identification of microbial composition, each of the three samples collected at the three time points from eight individuals were combined and analyzed. All sequences were found to be associated with 13 bacterial phyla and, in particular, within five phyla commonly encountered in the human intestine: Actinobacteria, Bacteroidetes, Firmicutes, Fusobacteria, and Proteobacteria [24], [25]. The Firmicutes and Bacteroidetes phyla predominated and together harbored on average 94.8% (SD = 3.9%) of the sequences. This is in agreement with previous studies attributing the majority of human gut microbiota to these two phyla [2], [38]. The average relative abundance of microorganisms from the Bacteroidetes and Firmicutes phyla was 24 (SD = 17.6%) and 70.8% (SD = 17.5%), respectively. We examined the relationship between gut microbial composition and age or obesity as previous studies have reported a higher proportion of Bacteriodetes in the elderly than in younger, healthy adults or infants [10], [41]. Other studies have also highlighted a higher Firmicutes to Bacteroidetes (F/B) ratio in obese than in lean individuals [42]. However, at the phylum level, the current study failed to find any correlation between the proportion of Bacteroidetes and age (Figure S5A; R^2^ = 0.018) or between the F/B ratio and body mass index (BMI) (Figure S5C; R^2^ = 0.011). Inferring the correlation between gut microbial composition and BMI or F/B ratio using all individuals is problematic because the gut microbial community analysis was carried out using combined data from three different time points in eight individuals (individuals A to H) and the composition of the community in each sample showed temporal variation within the same person. However, no relationship was found between these traits when the eight individuals that were sampled on three occasions were excluded (Figure S5B and D; R^2^<0.035). The variation in microbial composition within an individual is described in detail in the section ‘*Host specificity and temporal stability of gut microbiota’.*

**Figure 3:**
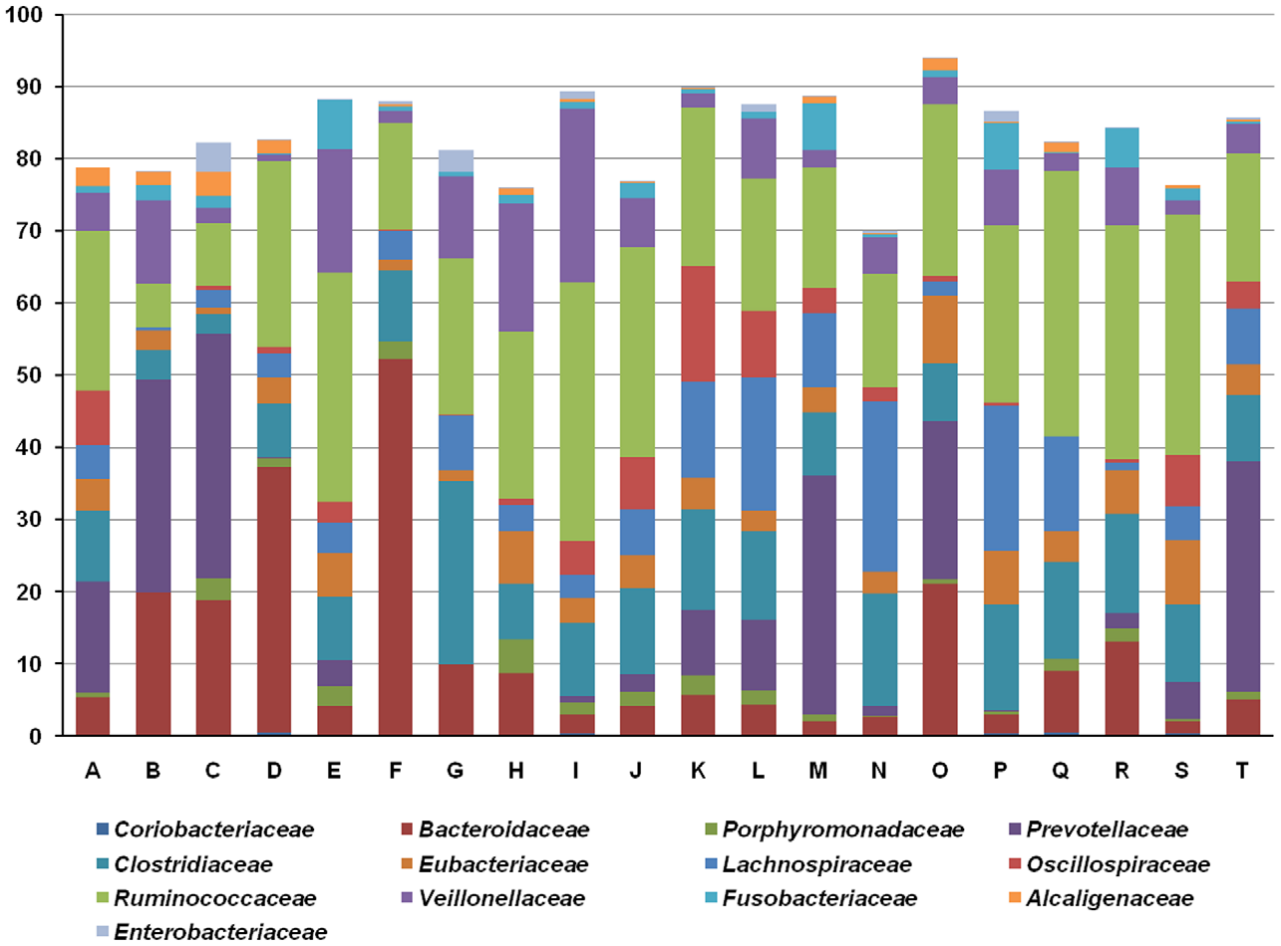
Composition of 13 major bacterial families in Korean gut microbiota. The abundance of each family member was determined with partial sequences of bacterial 16S rRNA genes from fecal samples of 20 Koreans using classifications by MOTHUR with a modified 16S rRNA database from the Ribosomal Database Project (RDP).

Figure 3 shows the relative abundance of 13 major family groups. An average of 56.7% (SD = 17.5%) of all sequences belonged to the 6 families comprising Firmicutes: Clostridiaceae, Eubacteriaceae, Lachnospiraceae, Oscillospiraceae, Ruminococcaceae, and Veillonellaceae. The three families of Bacteroidetes (Bacteroidaceae, Porphyromonadaceae, and Prevotellaceae) accounted for an average of 22.8% (SD = 17.8%) of sequences. Other families (Coriobacteriaceae, phylum Actinobacteria; Fusobacteriaceae, phylum Fusobacteria; Alcaligenaceae and Enterobacteriaceae, phylum Proteobacteria) accounted for less than 9.2% of all sequences. The most dominant family differed between individuals and the proportion of sequences attributable to the families Prevotellaceae and Ruminococcaceae showed the greatest between-individual variations of 12.7 and 8.5%, respectively. At the genus level, most sequences were distributed between 38 genera and, of these, 31 genera were members of the Bacteroidetes and Firmicutes phyla and accounted for 75.4% (SD = 6.3%) of the relative abundance (Table S2). Dethlefen *et al*. [33] have also reported that the majority of members of gut microbial communities belong to a small number of genera within the Bacteroidetes and Firmicutes phyla. The three most abundant genera were *Faecalibacterium*, *Prevotella*, and *Bacteroides*, which together accounted for an average of 30.2% of Korean gut microbiota. Members of the genus *Faecalibacterium* were the most numerous in these communities (average = 11.8%) and *F. prausnitzii* has been estimated to account for approximately 10% of human fecal bacteria [2]. The genus *Prevotella* contributed 9.5% of the microbial community. This genus contains carbohydrate- and protein-fermenting, acetate and H_2_-producing bacteria such as *P. ruminicola* [43]. Members of the genus *Bacteroides* include *B. thetaiotaomicron* and are known to be involved in nutrient absorption and epithelial cell maturation and maintenance [44]. Genus *Bacteroides* had an average abundance of 8.9%. As Figure S6 shows, the sequences identified in the study were distributed between few taxa above the genus level (average taxon at phylum level 9.15; family 32.6; genus 107). However, each person harbored an average of 771 species level phylotypes (SD = 313), which is constant with previous reports that the diversity of specific taxa at the phylum to genus levels is relatively low but is extremely high at the species and strain levels [33], [45].

### Host specificity and temporal stability of gut microbiota

**Figure 4:**
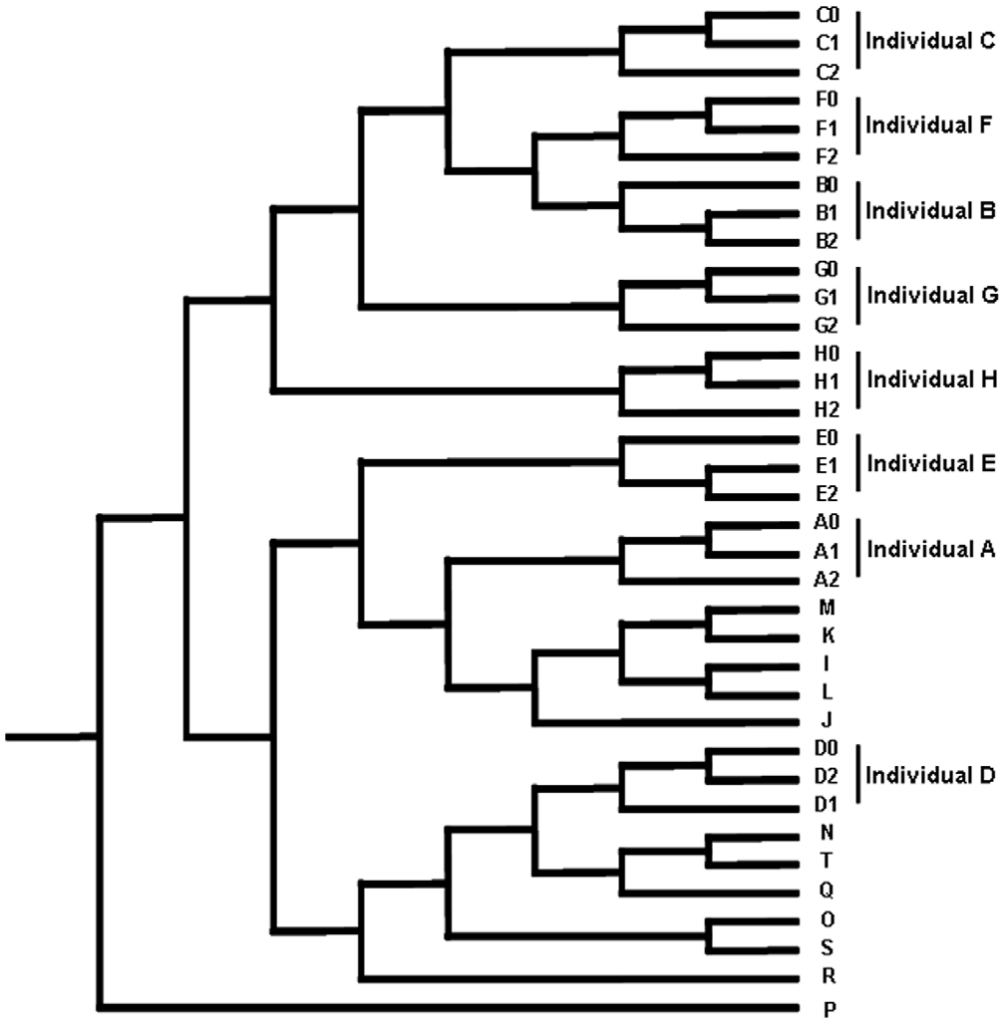
Comparison of the communities of gut microbiota in each sample. Distances between communities were calculated with the Jaccard coefficient and each community was clustered using the UPGMA algorithm. Numbers alongside the capital letters indicate the sampling period; 0 months, 1 month, or 2 months after the initial sampling.

**Figure 5:**
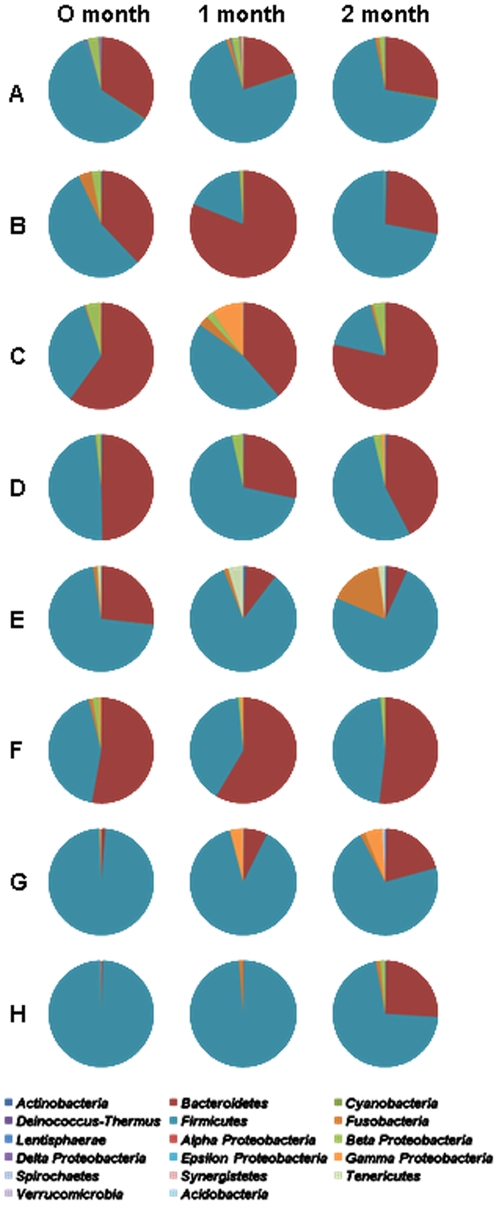
Temporal changes in gut microbial communities. Changes in the relative abundance of microbes within an individual were investigated using three samples collected from each of eight Koreans at monthly intervals. The relative abundance of partial sequences of the bacterial 16S rRNA gene were estimated by classification at the phylum level using MOTHUR with a modified 16S rRNA database from the Ribosomal Database Project (RDP).

**Figure 6:**
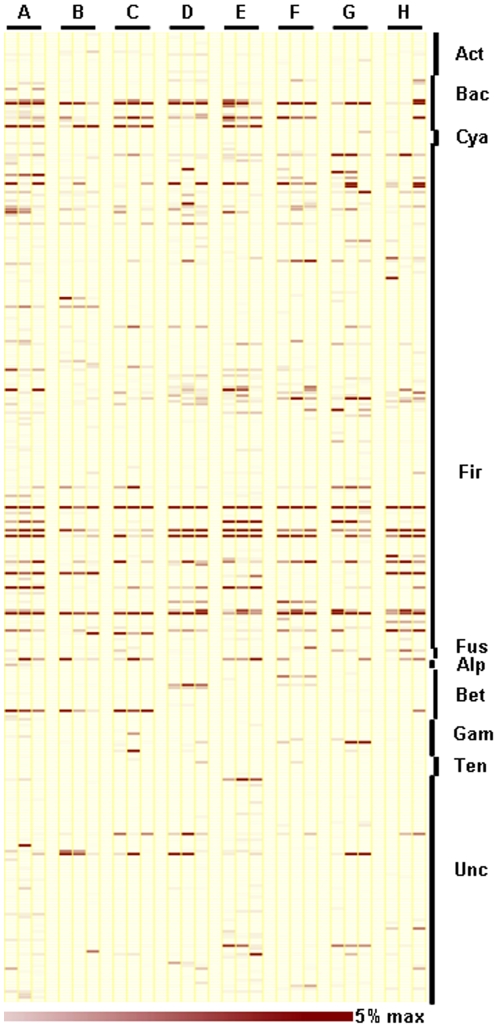
Changes in the pattern of gene taxa over the test periods. Each column in the heatmap represents a sample from eight individuals. Three samples from the same individuals were grouped together in parallel. Each row represents a genus. The color intensity of the panel is proportional to the abundance of OTUs (max 5%). The staggered bars on the right indicate the phyla (Act = *Actinobacteria*, Bac = *Bacteroidetes*, Cya = *Cyanobacteria*, Fir = *Firmicutes*, Fus = *Fusobacteria*, Alp = Alpha-*Proteobacteria*, Bet = Beta-*Proteobacteria*, Gam = Gamma-*Proteobacteria*, Ten = *Tenericutes*, Unc = Unclassified bacteria).

As shown in Figure 4, the samples from different people could be readily separated and the three samples collected from the same individuals clustered together, indicating that short term temporal changes in community structure within an individual were minor compared to inter-personal differences. These observations are consistent with previous studies reporting that gut microbial communities repeatedly sampled from the same individual show greater similarities than those from family members or unrelated individuals, and that microbial communities at two time points from the same individual vary in the relative abundance of major bacterial phyla but not in the actual phylotypes present [24]. The Jaccard coefficient applied to construct similarity matrices only reflected the community membership rather than the relative abundance of each member. Therefore, we can conclude that each individual harbors a large number of host-specific microbial phylotypes and those short-term temporal changes in the species composition of gut microbial communities within an individual are minor. As shown in Figure 5, the phylum level composition of gut microbiota in the eight individuals varied through the test period. In particular, the proportions of Bacteroidetes and Firmicutes in individuals B and C were somewhat disturbed. This can be evidenced by the deviations in the Bacteroidetes/Firmicutes ratio of 2.35 and 2.0 in these individuals, respectively, as compared to a deviation in other people of only 0.14 to 0.3. To compare community characteristics in greater detail, heat maps were constructed representing the relative abundance of each genus (Fig. 6). These clearly show that most members of the gut microbiota belonged to genera within the Bacteroidetes and Firmicutes phyla. Whilst each host-specific genera was present in each of the repeated samples, the relative abundance of many genera fluctuated through the test period. Furthermore, individuals largely possessed the same genera but the most variable of these differed between people. The most variable genera were *Prevotella* (individuals A, B, and C, deviation>11.5%), *Bacteroides* (individual D, deviation = 10.8), *Faecalibacterium* (individual E, deviation = 14.2), *Ruminococcus* (individual G, deviation = 7.5), *Fusobacterium* (individual E, deviation = 10.3), *Dialister* (individual G, deviation = 9.2), *Eubacterium* (individual H, deviation = 18.2), and *Megamonas* (individuals B and H, deviation>27.7). In addition, we plotted Shannon diversity indices of the three samples collected from the eight individuals to evaluate whether total gut microbial diversity altered over time. Diversity indices varied between individuals (average diversity in an individual = 3.77 to 5.91) and over time (SD = 0.09 to 0.53) (Figure S7). The Shannon diversity index accounts for both the richness and evenness of OTUs, such that the index can be increased by either having additional unique species or by having greater species evenness. These results together indicate that the microbiota of the human intestine is host-specific and temporally stable in community structure, but that the abundance of each community member fluctuates continuously.

### Core gut microbiota of Korean people

**Figure 7:**
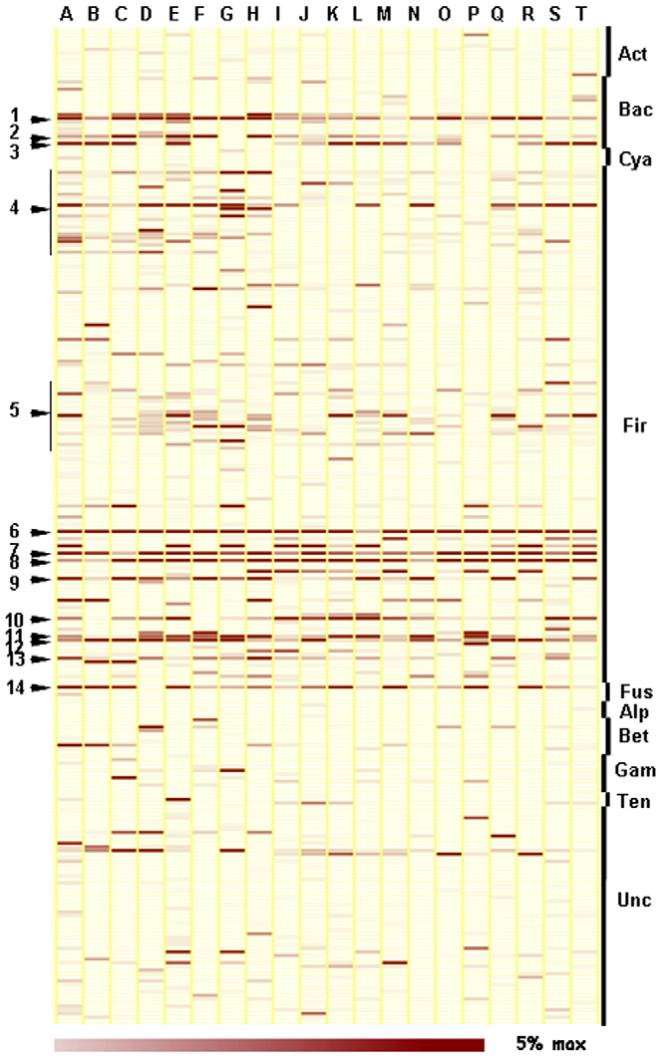
Relative abundance of taxa in all individuals studied. Each column in the heatmap represents one individual, whilst each row represents a genus. The color intensity of the panel is proportional to the abundance of OTUs (max 5%). The staggered bars on the right indicate the phyla (Act = *Actinobacteria*, Bac = *Bacteroidetes*, Cya = *Cyanobacteria*, Fir = *Firmicutes*, Fus = *Fusobacteria*, Alp = Alpha-*Proteobacteria*, Bet = Beta-*Proteobacteria*, Gam = Gamma-*Proteobacteria*, Ten = *Tenericutes*, Unc = Unclassified bacteria). The numbers on the left represent the genus of the predominant core gut microbiota candidates: 1. *Bacteroides*, 2. *Parabacteroides*, 3. *Prevotella*, 4. Uncultured butyrate-producing bacterial group of the *Firmicutes*, 5. Uncultured human intestinal *Firmicutes* group, 6. *Clostridium*, 7. *Eubacterium*, 8. *Faecalibacterium*, 9. *Lachnospira*, 10. *Oscillibacter*, 11. *Roseburia*, 12. *Ruminococcus*, 13. *Subdoligranulum*, and 14. *Fusobacterium*.

A major interest in research on human gut communities is to determine whether core microbiota exist that are broadly distributed between all or a vast majority of individuals. To identify core microbiota candidates present in Korean intestines, we constructed heat maps with pyrosequencing data at the genes level (Fig. 7). These candidates included *Bacteroides*, *Parabacteroides*, and *Provotella* in the *Bacteroidetes* phylum; *Clostridium*, *Eubacterium*, *Facalibacterium*, *Lachnospira*, *Oscillibacter*, *Roseburia*, *Ruminococcus*, *Subdoligranulum*, butyrate-producing bacteria and uncultivated human intestinal clones in the Firmicutes phylum; and *Fusobacterium* in the phylum Fusobacteria. Among these core genera, *Eubacterium*, *Roseburia* and *Faecalibacterium* are known to be related to butyrate production [46], [47]. With fine scale OTU analysis, we detected 43 core gut microbiota candidates from 8,642 OTUs that were represented in at least 15 out of 20 individuals (Table 2). Interestingly, 15 of the 43 core microbiota candidate OTUs were closely related to the dominant butyrate-producing bacterial genera mentioned above and accounted for an average of 11.5% (SD = 6.2%) of the microbiota in the individuals tested.

**Table 2:** Core gut microbiota candidates widely distributed between individuals.

Taxonomic rank				aNumber of OTU
Phylum	Class	Family	Species	
Bacteroidetes	Bacteroidia	Bacteroidaceae	*Bacteroides galacturonicus*	2
Bacteroidetes	Bacteroidia	Bacteroidaceae	*Bacteroides* sp. CO55	1
Bacteroidetes	Bacteroidia	Bacteroidaceae	*Bacteroides* sp. CS3	1
Bacteroidetes	Bacteroidia	Bacteroidaceae	*Bacteroides uniformis*	1
Bacteroidetes	Bacteroidia	Bacteroidaceae	*Bacteroides vulgatus*	2
Bacteroidetes	Bacteroidia	Porphyromonadaceae	*Parabacteroides merdae*	1
Firmicutes	Clostridia	Butyrate-producing bacterium	Butyrate-producing bacterium A1-86	1
Firmicutes	Clostridia	Butyrate-producing bacterium	Butyrate-producing bacterium A2-207	2
Firmicutes	Clostridia	Butyrate-producing bacterium	Butyrate-producing bacterium M21/2	3
Firmicutes	Clostridia	Butyrate-producing bacterium	Butyrate-producing bacterium SL6/1/1	1
Firmicutes	Clostridia	Butyrate-producing bacterium	Butyrate-producing bacterium SSC/2	1
Firmicutes	Clostridia	Butyrate-producing bacterium	Butyrate-producing bacterium T1-815	1
Firmicutes	Clostridia	Clostridiales bacterium	*Clostridiales* bacterium 80/4	1
Firmicutes	Clostridia	Clostridiaceae	*Clostridiaceae* bacterium DJF LS13	1
Firmicutes	Clostridia	Clostridiaceae	*Clostridium bolteae*	1
Firmicutes	Clostridia	Clostridiaceae	*Clostridium orbiscindens*	1
Firmicutes	Clostridia	Clostridiaceae	*Clostridium saccharolyticum*	1
Firmicutes	Clostridia	Eubacteriaceae	*Eubacterium tenue*	1
Firmicutes	Clostridia	Eubacteriaceae	*Eubacterium ventriosum*	1
Firmicutes	Clostridia	Ruminococcaceae	*Faecalibacterium prausnitzii*	4
Firmicutes	Clostridia	Ruminococcaceae	*Faecalibacterium* sp. DJF VR20	2
Firmicutes	Clostridia	Ruminococcaceae	*Ruminococcus gnavus*	1
Firmicutes	Clostridia	Ruminococcaceae	*Ruminococcus* sp. CB3	2
Firmicutes	Clostridia	Ruminococcaceae	*Ruminococcus* sp. CJ60	2
Firmicutes	Clostridia	Ruminococcaceae	*Ruminococcus* sp. K-1	1
Firmicutes	Clostridia	Ruminococcaceae	*Ruminococcus* sp. SC103	1
Firmicutes	Clostridia	Ruminococcaceae	*Subdoligranulum* sp. DJF VR33k2	1
Firmicutes	Unclassified	Unclassified	*Firmicutes* bacterium EG20	1
Unclassified	Unclassified	Unclassified	Human intestinal bacterium PUE	1
Unclassified	Unclassified	Unclassified	Human intestinal firmicute CO35	2
Unclassified	Unclassified	Unclassified	Rumen bacterium 8/9293-21	1

a A species level OTU was regarded as a core gut candidate when present in more than 15 of the 20 individuals.

### Comparison of gut microbiota between Korean and non-Korean peoples

**Figure 8:**
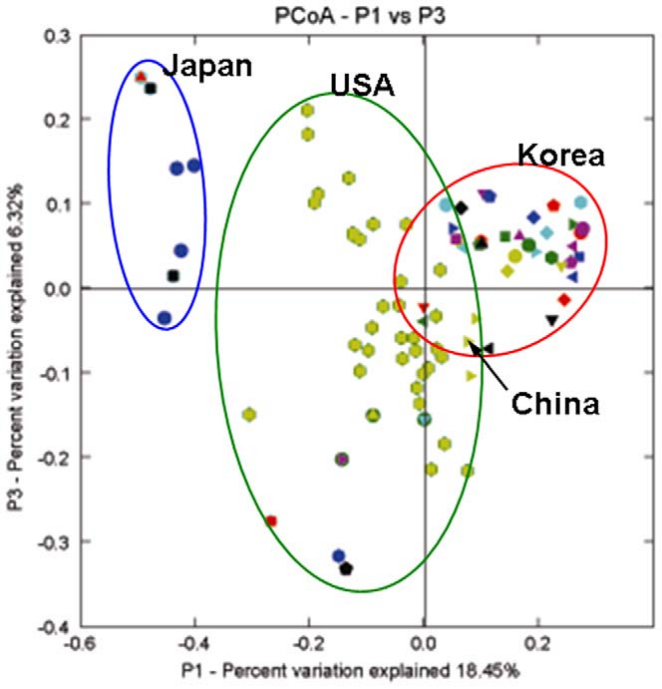
Comparison of Korean gut microbial communities to that of non-Korean people. The plot was generated using an un-weighted UniFrac analysis. The large green, blue circles and black arrow represent the US and Japanese clusters, respectively. Almost all of the Koreans sampled converged into the large red circle. Reference sequence data of US, Japanese and Chinese individuals were aligned with the sequences of Korean gut microbiota and trimmed to match the same position and length of the 16S rRNA gene.

To compare the overall compositions of Korean gut microbial communities with those of people from the USA, China, and Japan, 16S rRNA gene sequences spanning the same region as those in our dataset were examined. Analysis was performed using Fast UniFrac [48], which measures the similarity between bacterial communities based on phylogenetic distances. Previously reported 16S rDNA gene sequence data for each person were truncated to match the length and position of the Korean gut 16S rDNA sequences and then all the sequences were processed at the same length and position. All pair-wise distances between communities were computed and Principal Coordinate Analysis (PCoA) was used to cluster the communities along axes of maximal variance (Fig. 8). Gut microbial community of each country member was roughly clustered by country and the maximum variations were 18.45% (PC1) and 6.32% (PC3). While the samples from Korea, the USA, and Japan segregated on PC1, some individuals from each country having intermediate values. US and Japanese samples were broadly distributed along PC3 but Korean samples were relatively converged into a narrow position.

For the comparison of gut microbial community composition among each country member, microbial sequence data from each individual in same country were combined into one representative dataset and then gut microbial community composition of four countries was analyzed. As shown in Figure S8, gut microbial community of each country member was comprised of three major phyla including Firmicutes, Bacteroidetes and Actinobacteria but the abundances of each phylum were slightly different from each other; American had higher Firmicutes (61.0%) than other countries (<55.6%), Japanese had higher Actinobacteria (22.1%) than other countries (<2.5%), and Korean and Chinese showed Bacterodetes-rich gut microbiota (30.2 and 36.6%, respectively) than other countries (<18.2%). In genus level diversity (Table S3), Japanese showed higher abundance of genus *Bifidobacterium* (20.6%, compared with other countries showing <1.8%) and *Clostridium* (10.3%, other countries <4.5%). Chinese showed the highest abundance in a genus *Bacteroides* (30.3%, other countries <12.9%) and lowest abundance in a genus *Clostridium* (0.8%, other countries>4.0%). In the other hand, Korean showed relatively higher abundance in the genus *Prevotella* (10.5%) and *Faecalibacterium* (11.6%), compared with other countries showing <0.5 and <9.4%, respectively

## Discussion

The human intestine, containing more than 5,000 bacterial species, is one of the most densely populated microbial habitats on Earth [33]. These organisms have a number of beneficial effects on nutrition, immune competence, and cell development in the host [5], [7]. Colonization by gut microbiota begins at birth and wide-ranging factors such as diet, geographical region, and, above all, host genotype are related to the characteristics of the microbial community that develops [34], [49]. Once established, these host-specific microbial communities generally remain stable throughout life [50], which has allowed the conduct of a large number of studies characterizing human gut microbiota and the relationship between dysbiosis and diseases such as IBDs and cancers [9], [10], [11]. However, previous studies investigating gut microbial composition have been biased towards sampling people from the west and some Asian countries [51], [52], and little is known about Korean gut microbiota. In addition, to our knowledge, no related studies targeting Korean individuals with deep sequencing methodology have been published so far. To address this lack of information, the gut microbiota of 36 samples from 20 Koreans were analyzed in the present study using pyrosequencing of the V1/V2 region of the 16S rRNA gene.

We analyzed 303,402 sequence reads and detected more than 8,600 phylotypes with the 97% similarity cut-off value from all V1/V2 sequences. This level of diversity is considerably higher than that reported in previous studies. For example, Cleasson *et al.* [38] detected around 5,200 phylotypes from 740,704 pooled V4 sequences of four individuals whilst McKenna *et al.* [37] identified around 5,000 OTUs from 141,000 pyrosequencing reads of concatenated V1 and V2 sequences from 12 macaques. The composition of gut microbiota varies greatly between people and only a small fraction of microorganisms are shared between two individuals, even where these are family members [24]. Moreover, even genetically identical twins have a different composition of gut microbiota, although the communities share more similarities than those of unrelated individuals [24], [34]. The sequence sample size analyzed by McKenna *et al.* was smaller than that of Cleasson *et al*. but a similar number of phylotypes was identified as the former study investigated three times more subjects than the latter. These reports indicate the existence of high host-specificity in the composition of gut microbial populations.

In our study, 4,706 of a total of 8,642 species level phylotypes were concatenated by single or double sequences. Although these species comprising the rare biosphere may only be present in small numbers, they may still serve as keystone organisms within that community with the potential to proliferate when environmental conditions become more favorable to their growth [40]. Furthermore, they may act as environmental gene reservoirs for reshaping the genomic constitution of other microorganisms in their community through horizontal gene transfer (HGT) [48]. Consequently, in the present trial, a great diversity of rare OTUs seems to have been detected from the rare biospheres of the Korean samples and future investigations using high throughput deep sequencing analysis targeting various subjects will be required to precisely understand the evolutionary function of these organisms in humans.

Our deep sequencing analysis revealed that most of the identified microorganisms were members of the Bacteroidetes and Firmicutes phyla, and that the latter constituted approximately 70% of the total community. Conversely, relatively few sequences were associated with the Proteobacteria, Fusobacteria, and Actinobacteria phyla, which together contributed less than 5% to the total community. Therefore, at the phylum level, the community composition of Korean gut microbiota seems to be similar to that previously reported for other human populations [1], [2]. Interestingly, when Gill *et al.* [53] used the qPCR method, they found only a small proportion of microorganisms belonging to the Bacteroidetes phyla, but when 237 partial 16S rDNA sequences derived from metagenomic data and 2,062 near full-length 16S rDNA gene sequences were analyzed, the bacterial phylotypes were assigned to only two divisions (Firmicutes and Actinobacteria). In our study, eight out of the 20 individuals also possessed a high number of Firmicutes, which accounted for over 80% of the relative abundance. However, we found no relationship between the phylum level community composition and either age or obesity status. The proliferation of the Firmicutes phylum seems therefore to be a general phenomenon of host-specific gut ecosystems.

Variations in rRNA gene copy numbers may provide another possible reason for the large difference in microbial composition accounted for by the two major phyla (Bacteroidetes and Firmicutes). Natural selection and evolutionary forces have shaped the size of genes favoring the elimination of redundancy in prokaryotes, but multiple copy numbers of rRNA genes are easily found in a broad range of microorganisms. Variations in rRNA gene copy numbers reflect differences in survival strategies between microorganisms, with those possessing a greater number of rRNA gene copies typically being more able to rapidly respond to environmental changes that encourage their growth [54]. Therefore, it is important to consider the 16S rRNA gene copy number when estimating the size of bacterial populations in addition to simply counting the number of individual sequences. It is known that the average copy numbers of the 16S rRNA gene in microorganisms belonging to the Bacteroidetes and Firmicutes phyla is 3.68 and 6.35, respectively, when calculated using currently available microorganisms at rrnDB (http://ribosome.mmg.msu.edu/rrndb/index.php). However, these estimates are based on 22 and 203 microbial strains per respective phylum, and additional data would benefit the more accurate estimation of gene copy numbers, particularly in the Bacteroidetes phylum. Until this occurs, discrepancies in 16S rRNA gene copy number between microorganisms might affect our interpretation of the composition of the actual microbial communities present.

Despite the temporal fluctuation seen in the present study in the host-specific F/B ratio, when Firmicutes and Bacteroidetes were taken together, their contribution to the total microbial ecosystem remained largely constant over time and between individuals (average = 94.8%; SD = 3.9%). Lozupone *et al.* [55] has previously described how phylogenetically disparate microorganisms that share a habitat may converge in their genetic constitution as they adapt to confront the same challenges. Turnbaugh *et al.* [24] has also reported that specific bacterial phylotypes do not exist in all tested individuals, but the relative abundance of broad functional categories of genes related to carbohydrate and amino acid metabolism are generally consistent regardless of the sample surveyed. Therefore, it is expected that many members of the Bacteroidetes and Firmicutes phyla possess the same functional genes. Similarly, the overall microbial gene pools present in a human gut may remain largely constant throughout life, while the microbes themselves are continuously replaced over the short term in response to environmental change.

Interestingly, we detected 43 core bacterial phylotype candidates present in at least 15 of the 20 Koreans tested and, of these, 15 candidates are related to butyrate-producing bacteria. In the human intestine, small chain fatty acids (SCFAs), such as acetate, propionate and butyrate, are produced by groups of gut microbiota mainly from dietary fibers (plant polysaccharides) [56]. Korean takes higher dietary fiber (19.8 g/day) than American (15.1 g/day) or Japanese (15.0 g/day) and the main source of dietary fibers comes from vegetables and cereals including kimchi and steamed rice (65.71% of total dietary fiber intake) [57]. SCFAs, especially butyrate, have an important role in strengthening the function of the gut mucosal barrier by stimulating the growth of mucosal cells. For example, colonocytes absorb SCFAs as their principal energy source and butyrate accounts for 70% of their oxygen consumption [58]. Diminished butyrate oxidation may lead to breaches in the colonic epithelial cell barrier and subsequent collapse of the defense capability, causing intestinal diseases such as ulcerative colitis and Crohn's disease [59], [60]. Using metagenomic analysis, Gill *et al* [53] have also reported that butyrate kinase-related COG is the most enriched in the human gut microbiome.

The microbial community of human intestine being established after birth is determined by several factors such as host genetics, birth delivery mode, diet, pre- and probiotics. Among them, the effect of host genotype and diet has been largely investigated and their potentials capable of molding gut microbiota are well known. Zoetendal *et al*. [34] investigated relationships between genetic relatedness and microbial community of human intestine and found that monozygotic twins had higher similarities in microbial communities than dizygotic twins or unrelated individuals. However, when Turnbaugh *et al.* [24] analyzed the gut microbiota of pairs of twins along with their mothers and found no significant difference in the degree of similarity in gut populations between adult monozygotic twin pairs compared to dizygotic twin pairs. It suggests that some other factors along with host genotype are likely to contribute to shaping the composition of microbial communities. De Filippo *et al.* [49] reported that high sugar, animal fat, and calorie-dense diets can limit the adaptive potential of microbial populations. Furthermore, Hehemann *et al.* [61] revealed that the diet can induce HGT between microorganisms in the genus *Bacteroides* in Japanese individuals consuming algae in their diet.

The gut microbial communities of Koreans in our study did show inter-individual differences but, when compared with people from other countries, microbial community of each county member sharing similar diet style and genotype roughly grouped together and the difference between Korean individuals was relatively smaller compared to the other county members.

Modern science applies more effective analysis techniques to the description of gut microbiota than was possible in the past, but a full understanding of human gut communities still remains elusive. Intensive sequencing analysis such as 454 pyrosequencing offers the ability to assess many sequences at low cost. As a single pyrosequencing run produces over 1,000,000 reads, we can now use this method to analyze a microbial community at a level of detail that is orders of magnitude greater than is possible with traditional culture-based or Sanger sequencing approaches. Despite this, even such detailed analysis is insufficient to fully understand the characteristics of human gut microbiota. This reflects the fact that individuals predominantly harbor members of a small number of common species, few of which are present in all people, whilst each of us possesses hundreds of rarer microbes. Moreover, the ability of intensive sequencing methods to accurately assign taxonomic rank is still lower than that of traditional sequencing approaches using full-length 16S rRNA gene sequences. However, the gap in the sequence read length between the products of pyrosequencing and traditional sequencing has been gradually reduced and will disappear completely in the near future as a result of continuous improvements in technology.

While 16S rRNA gene based analysis are advantageous for describing the human gut microbial diversity and elucidating possible links between gut microbiota and health, diet, or host genotypes, potential functions of gut microbiota on the internal or external factors cannot be identified with community data. Therefore, other approaches not only examining microbial community by analysis of marker genes but also being able to identifying potential roles of microbes in the human gut are needed. Gill *et al.* firstly demonstrated that gut microbiota played beneficial roles in the human host by regulating many genes related to the metabolism of glycans, amino acids, biosynthesis of vitamins and isoprenoids by metagenome analysis [53]. More recently, Qin *et al.* analyzed fecal metagenome sequences of 124 European, identified 3.3 millions of microbial genes, and showed that each individual harbored 204,056 common genes [62]. While these metagenomic approaches gave us great insight to the functional possibility of gut microbiota to human host, it is not also be able to reflect the actual metabolic activity responding to various factors because it cannot differentiate between expressed and non-expressed genes. Therefore, metatranscriptome analysis of human gut microbiota differing in genetics, diet choice, and geographical region, and comparison of neonatal microbial communities with host genetics will be needed to fully understand the overall structure and function of gut microbiota in the future.

In addition, in this study, we incorporated only the reference sequences covering the same region of 16S rRNA genes that we analyzed but there might be inescapable bias originated from primer efficiency or analysis methods. All the sequences that we used as references and derived from our study used same forward primer but different reverse primers except Japanese individuals (Table S4). And the bacterial taxon coverage of each primer pairs were practically determined by forward primers because all reverse primers showed greatly higher coverage than forward primers except one study [35] when analyzed by RDP probe match (http://rdp.cme.msu.edu/probematch). We and Dethlefsen et al. [35] used primers containing one degeneracy and the degenerated primer showed 23.6% higher coverage compared to non-degenerated primer. Moreover, appearance of higher abundance of Bifidobacteria in Japanese samples was possibly occurred by metagenomic analysis because forward primer used in this study could not amplify Bifidobacteria by three base sequence mismatches. Therefore, in the future studies, methodological unification will be needed because comparision with reference data sets generated with different methods of DNA extraction, PCR, and sequencing chemistry might have potential biases in microbial community analysis.

In this study, we investigated gut microbiota of 20 Korean individuals and identified more than 8,600 species level phylotypes. Korean gut microbiota showed host specificity, but the abundance of each community members fluctuated during test period. While overall feature of Korean gut microbiota did not differ from that of other country members of American, Chinese and Japanese above genus level phylotypes, fecal microbial community of each country member showed slight difference from each other. In addition, we identified core gut microorganisms widely distributed in Korean individuals and many of these are related to butyrate-producing bacteria suggesting that diet style might affect in molding gut microbiota as well as host genetics.

## Materials and Methods

### Sampling and DNA extraction

Twenty healthy individuals who had not taken antibiotics within the previous year were recruited to donate stool samples. Informed consent was obtained from all participants and the study protocol was approved by the Institutional Review Boards of Kyung Hee University (KHU IRB 2010–008). Three stool samples were collected at one month intervals from eight of the individuals and a single stool sample was collected from the remaining twelve. Approximately 5 g of stool sample was collected into sterile plastic containers by the participants themselves and immediately stored in home freezers until brought to the experimental laboratory. The samples were stored at -80°C until further processing. Participant characteristics and the sampling regime are shown in Table 3.

**Table 3:** Samples of GI microbiota analyzed.

Individual	Sample ID	Number of samples	Age	BMI	Gender
Individual A	A0	Three samples collected with one month interval between each	36	21.6	Male
	A1				
	A2				
Individual B	B0		31	22.5	Male
	B1				
	B2				
Individual C	C0		28	21.6	Male
	C1				
	C2				
Individual D	D0		29	21.3	Male
	D1				
	D2				
Individual E	E0		31	22.0	Male
	E1				
	E2				
Individual F	F0		40	29.2	Male
	F1				
	F2				
Individual G	G0		6	20.7	Female
	G1				
	G2				
aIndividual H	H0		4	24.7	Female
	H1				
	H2				
Individual I	I	Single sample	37	21.8	Female
Individual J	J		64	24.0	Male
Individual K	K		63	22.7	Female
Individual L	L		63	24.4	Female
Individual M	M		66	27.8	Female
Individual N	N		64	24.1	Female
Individual O	O		64	23.9	Female
Individual P	P		60	28.7	Male
Individual Q	Q		68	28.2	Male
Individual R	R		32	23.1	Male
Individual S	S		28	21.2	Male
Individual T	T		27	21.2	Male

a Individual H: this person was the daughter of individual A.

A subsample of approximately 1 g of each stool sample was fully homogenized with 0.5 mm diameter glass beads (Roth, Karlsruhe, Germany) and liquid nitrogen and transferred into a sterile 15 mL conical tube. The homogenized samples were kept on ice until the addition of 5 mL of STES buffer (0.5 M NaCl, 0.2 M Tris-HCl (pH 7.6), 0.01 M EDTA, 1% SDS) and incubated for one hour at 60°C. DNA was subsequently extracted and purified with standard phenol/chloroform and ethanol precipitation methods [63]. Precipitated DNA was re-suspended in deionized water and the purity was improved using the UltraClean Microbial DNA Isolation Kit (Mo Bio Laboratories, Calsbad, CA, USA). The DNA concentration and quality was determined by agarose gel electrophoresis (1% wt/vol agarose in Tris-acetate-EDTA (TAE) buffer) and with a NanoDrop ND-1000 spectrophotometer (NanoDrop Technologies, Wilmington, DE, USA).

### Pyrosequencing of bacterial 16S rRNA fragments

To amplify the 16S rRNA gene fragments, 30 ng of purified DNA (determined by a NanoDrop 1000 spectrophotometer) served as a template in 30 µL reactions of a PCR premix solution containing 1.25 U Ex Taq DNA polymerase, 5 µL 10 X Ex Taq buffer, 3 mM MgCl_2_, 0.2 mM dNTP mix (SolGent, Daejeon, Korea). The V1 to V3 hyper-variable regions of the bacterial 16S rRNA gene [64] were amplified with the primer pair V1-9F (5′-X-AC-GAGTTTGATCMTGGCTCAG-3′) and V3-541R (5′-X-AC-WTTACCGCGGCTGCTGG-3′). The primers contained four to eight base sample-specific barcode sequences denoted as ‘X’ and common linker (AC) sequences in the 5′ end [65]. This approach allowed the analysis of PCR products from multiple samples in parallel on a single 454 picotiter plate [66] and an ability to re-sort the sequences into order. To minimize PCR bias where highly represented microorganisms are amplified faster than those that are rare, we used 18 PCR cycles for the 16s rRNA gene fragment amplicon. Thermocycling was conducted in a C 1000 Thermal Cycler (Bio-Rad, Hercules, CA, USA) under the following condition: initial denaturation at 94°C for 2 min; 18 cycles of denaturation at 94°C for 30 sec, annealing at 55°C for 30 sec, and extension at 72°C for 1 min; and a final extension at 72°C for 10 min.

After the PCR reaction, the quality of the amplified PCR products (approximate length: 520 nt) was confirmed by electrophoresis with 1 µL of the PCR reaction mixture in 1% agarose gel (0.5X TAE buffer) and purified using the QIAquick PCR Purification kit (Qiagen, Valencia, CA, USA). An equal quantity (100 ng) of each PCR amplicon tagged with the sample-specific barcode sequences was pooled to give a total amount of 3.6 µg. The DNA concentration and quality were further assessed on a BioAnalyzer 2100 microfluidics device (Agilent, Santa Clara, CA, USA) using a DNA1000 lab chip (Agilent, Santa Clara, CA, USA) before the 454 pyrosequencing reaction. Finally, the pooled DNA was amplified by emulsion PCR before sequencing by synthesis using the massively parallel pyrosequencing protocol (Margulies *et al.*, 2005). Sequencing was performed through a 454 pyrosequencing Genome Sequencer FLX Titanium (Life Sciences, Branford, CT, USA) according to the manufacturer's instructions by a sequencing provider (Macrogen, Seoul, Korea).

### Sequence processing

The sequences generated from pyrosequencing were mainly analyzed with the software MOTHUR for identification of operational taxonomic unit (OTU), taxonomic assignment, community comparison, and statistical analysis [67]. Experimental sequences were firstly processed using the *trim.seqs* script. This filtered the data to minimize the effects of poor sequence quality and sequencing errors by removing sequences with more than one ambiguous base call and retaining only sequences that were 250 nt or longer. The program was used to search barcode sequences tagged to each sample, bin each sequence accordingly, and to scan each binned sequence for the trimming of the 16S forward primer sequence. As a result of this processing, sequences that were shorter than 250 nt, had one or more ambiguous base calls, or had multiple barcode or primer motifs were excluded from the analysis. We included only sequences with the forward primer motif to ensure that the highly informative V1/V2 region was available for taxonomic assignment. The sequences obtained in this study were uploaded and made available through the DDJB database under Project ID 60507.

### Operational taxonomic unit (OTU) determination and taxonomic classification

The trimmed sequences from each barcode bin were aligned using Infernal and associated covariance models obtained from the Ribosomal Database Project Group [68]. The aligned sequences based on secondary structural information were further trimmed to encompass the V1/V2 regions. This allowed accurate analysis using the same regions and simultaneously increased the alignment speed. Sequences spanning the same region with a similar length were realigned using the *align.seqs* script of MOTHUR with the SILVA compatible alignment database that contains 50,000 columns long and aligned 14,956 bacterial sequences (http://www.mothur.org/w/images/9/98/Silva.bacteria.zip). A distance matrix was calculated from the aligned sequences using the *dist.seqs* script and operational taxonomical units (OTUs; 90 to 100% sequence similarity) were assigned by *cluster* script using the furthest neighbor clustering algorithm.

The OTUs defined by a 3% distance level were phylogenetically classified using *classify.otu* script with a modified bacterial RDP II database containing 164,517 almost full-length 16S rRNA sequences prepared using TaxCollector (http://www.microgator.org) and a taxonomy file containing the complete taxonomic information of each sequence in the database from domain to species by 51% confidence threshold. The closest bacterial relative was assigned to each sequence corresponding to the best match in the database.

### Community comparison analysis

To examine the temporal stability of the microbial community within a single individual, pyrosequencing reads from each sample were assigned as an OTU with 97% sequence identity. The OTU information from each sample was then transferred into a dendrogram with the *tree.shared* script of MOTHUR. The distances between microbial communities from each sample (three samples from each of eight individuals and one sample from each of 12 individuals) were calculated using the Jaccard coefficient and represented as an Unweighted Pair Group Method with Arithmetic Mean (UPGMA) clustering tree describing the dissimilarity (1-similarity) between multiple samples. A Newick-formatted tree file was generated through this analysis.

Fast UniFrac [48] analysis was also performed to compare the human gut microbial communities of Koreans with those of people from the USA, China and Japan based on phylogenetic information. For this analysis, a megablast search and the phylogenetic distribution were evaluated with the Greengenes core data set (http://128.138.212.43/fastunifrac/download/reference_trees/GreenGenesCore-May09.ref.tre.gz) as the source of reference sequences and the reference tree. The Fast UniFrac analysis provides a measure of the similarity between communities by marking the sequences from each sample on a reference phylogenetic tree and then computing the fraction of the branch length on the tree unique to each sample. The Cluster Samples option was used to perform hierarchical clustering analysis using the un-weighted algorithm.

### Calculation of species richness and diversity indices

Shannon's diversity (H' = −∑*p*_i_ln(*p*_i_) where *p*_i_ is the proportion of taxon i) [69], ACE and Chao I richness indices [70], and rarefaction curves [71] were generated using the MOTHUR program. The 3% dissimilarity cutoff value was used for assigning an OTU. Good's coverage was calculated as G = 1−n/N, where n is the number of singleton phylotypes and N is the total number of sequences in the sample.

### Public data used

The 16S rRNA gene sequence data used in this study included the following (unless otherwise stated, sequences were downloaded from NCBI): (1) sequences of three US individuals with the accession AY984124-AY986384 [2]; (2) sequences from one Chinese and two US individuals with the accession EU764594-EU768801 [33]; sequences from 30 US individuals with the accession FJ362604-FJ372382 [24]; (4) sequences from six US individuals with accession FJ452085-FJ454865 [35]; and (5) 16S rRNA gene sequences retrieved from human gut metagenomic data from 13 Japanese individuals, which were downloaded from P. Bork's group at EMBL (http://www.bork.embl.de) [52].

## Supporting Information

Figure S1 The number of operational taxonomic units (OTUs) present in the full set of pyrosequencing reads was determined with various percentage identity thresholds. The x-axis shows the percentage identity and the y-axis represents the number of OTUs detected.
(DOCX)

Figure S2 The number of unique sequences present in the pyrosequencing reads (A) and the number of OTUs (B) plotted against the number of unique sequences from each individual.
(DOCX)

Figure S3 Rank-abundance curve for the total bacterial community present in Korean fecal samples constructed using OTUs with a 97% sequence identity. The most abundant OTUs detected over 500 times have been excluded from panel to shorten the y-axis.
(DOCX)

Figure S4 Rarefaction curves using the Shannon diversity index to estimate the diversity of taxa present in individual fecal samples of Koreans.
(DOCX)

Figure S5 Correlation between age or BMI and bacterial estimates by pyrosequencing. The correlation between age and the relative abundance of *Bacteroidetes* in all individuals is shown in (A) and the combined data excluding 12 individuals are shown in (B). The correlation between body mass index (BMI) and the ratio of *Firmicutes* to *Bacteroidetes* in all individuals is shown in (C), whilst the combined data excluding 12 individuals are shown in (D). Bacterial abundances were determined by pyrosequencing of the V1/V2 region of the 16S rRNA gene from 20 Koreans.
(DOCX)

Figure S6 Distribution of taxa in each individual at different taxonomic levels. Black bars represent the number of phylum level taxa detected in each individual. The grey and dark grey bars represent family level and genus level taxa, respectively.
(DOCX)

Figure S7 Variation in Shannon diversity index. Diversity indices of three samples from each of eight individuals are shown together with the average index of the three samples with SD.
(DOCX)

Figure S8 Relative abundance of gut microbiota analyzed with MOTHUR from combined dataset of members of four countries at the phylum level.
(DOCX)

Table S1 The number of operational taxonomic units (OTUs) in each sample determined with various percentage identity thresholds.
(DOCX)

Table S2 Relative abundance (%) of 38 genera from each individual.
(DOCX)

Table S3 List of gut microbiota detected with more than 1% abundance in members of four countries.
(DOCX)

Table S4 Primers used in this study and references for the amplification of human gut bacterial 16S rRNA genes.
(DOCX)
