# Supplementary figures and images for: Comparative Analysis of Korean Human Gut Microbiota by Barcoded Pyrosequencing
Source: PLoS One. 2011 Jul 29;6(7):e22109. doi: 10.1371/journal.pone.0022109 (PMC3146482; doi:10.1371/journal.pone.0022109)

Figure S1


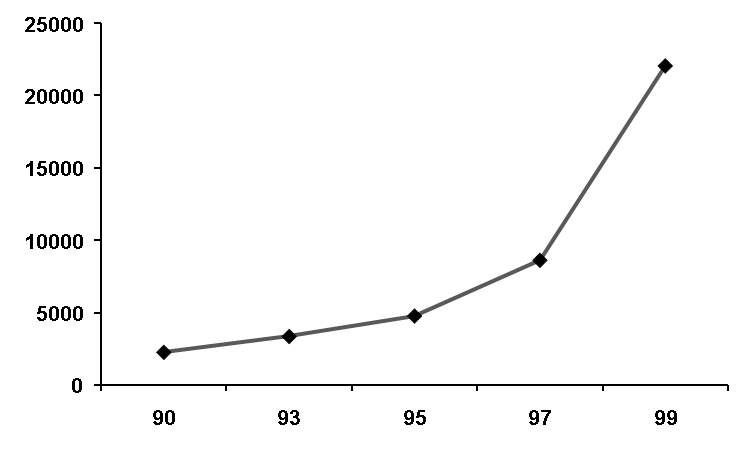

Supplement: Figure S1 — The number of operational taxonomic units (OTUs) present in the full set of pyrosequencing reads was determined with various percentage identity thresholds. The x-axis shows the percentage identity and the y-axis represents the number of OTUs detected. (DOCX) [file pone.0022109.s001.docx]

Figure S2


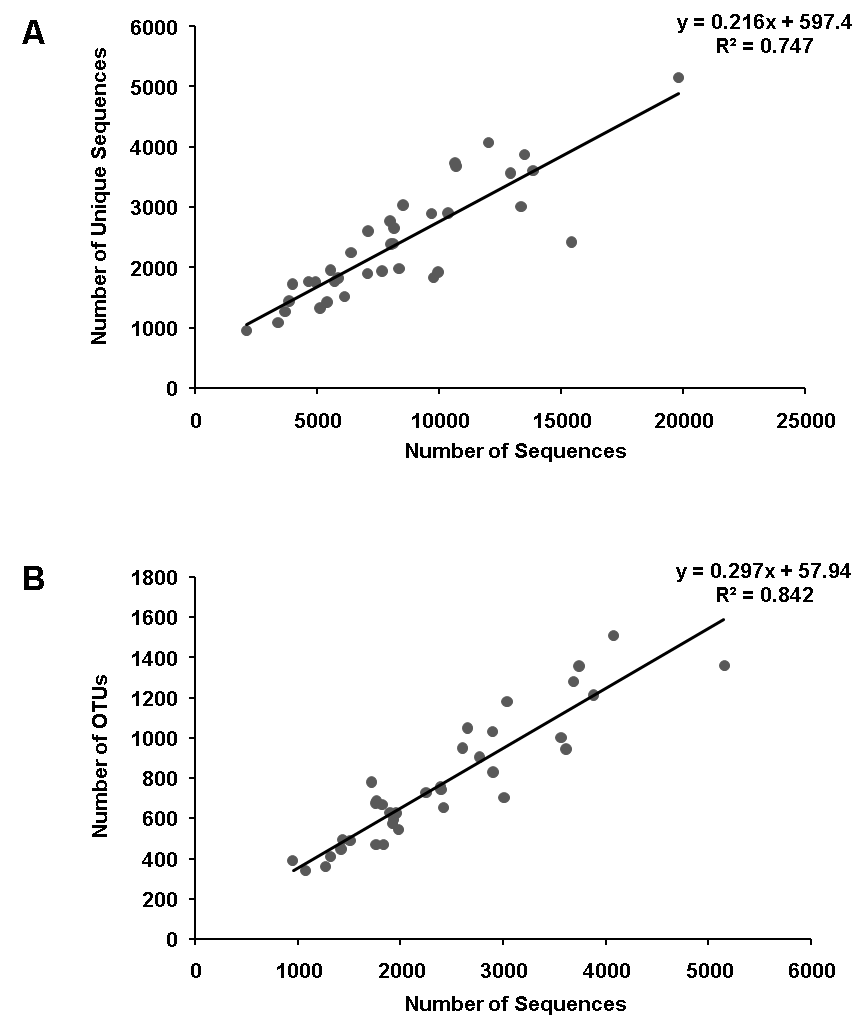

Supplement: Figure S2 — The number of unique sequences present in the pyrosequencing reads (A) and the number of OTUs (B) plotted against the number of unique sequences from each individual. (DOCX) [file pone.0022109.s002.docx]

Figure S3


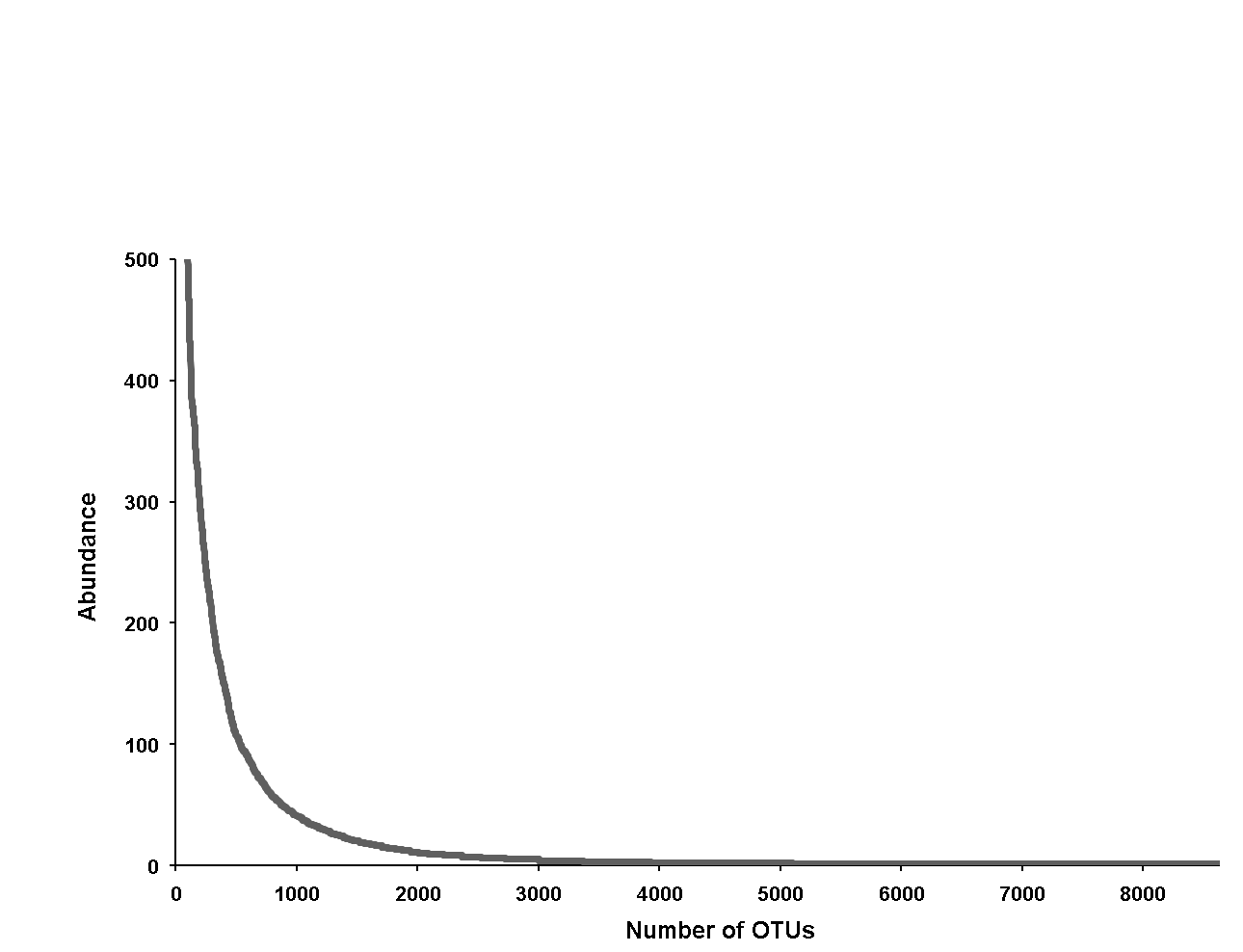

Supplement: Figure S3 — Rank-abundance curve for the total bacterial community present in Korean fecal samples constructed using OTUs with a 97% sequence identity. The most abundant OTUs detected over 500 times have been excluded from panel to shorten the y-axis. (DOCX) [file pone.0022109.s003.docx]

Figure S4


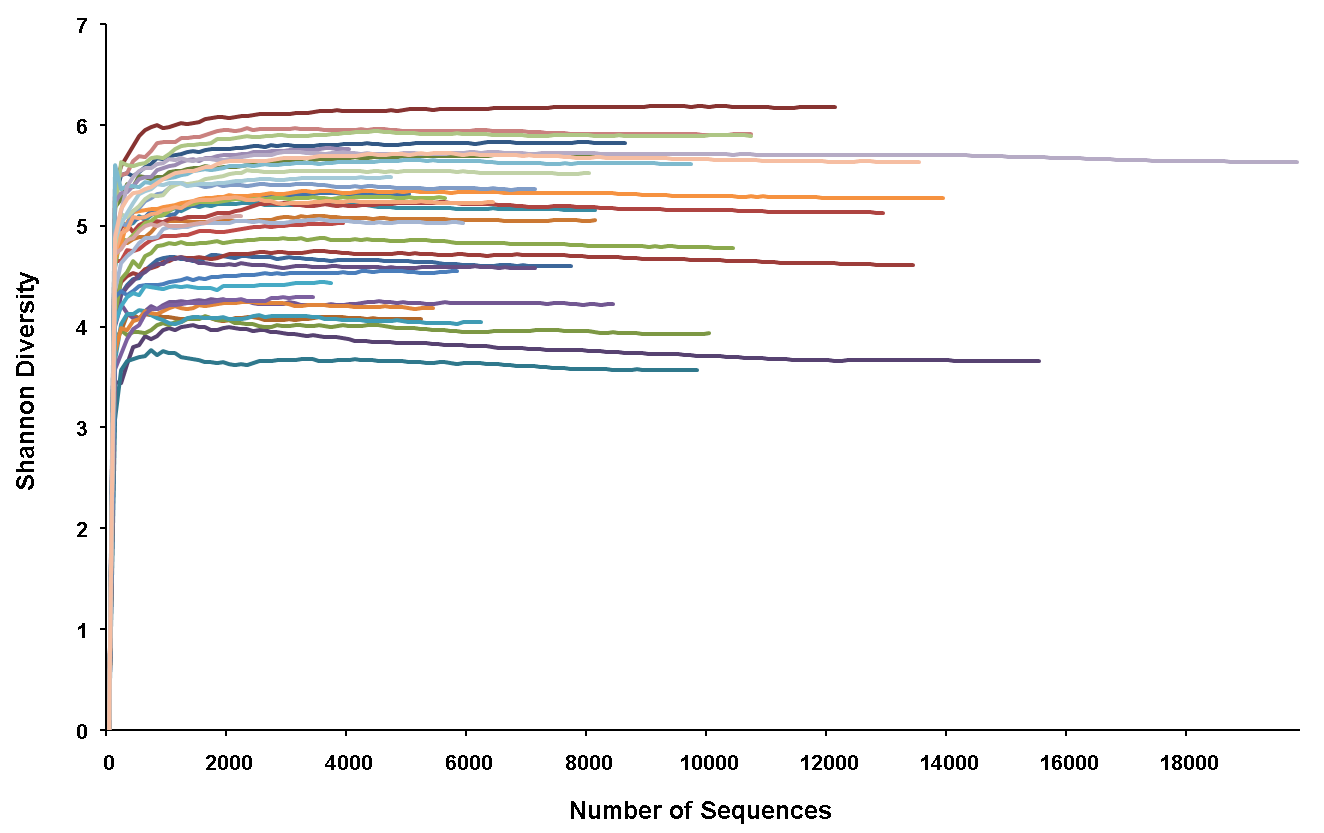

Supplement: Figure S4 — Rarefaction curves using the Shannon diversity index to estimate the diversity of taxa present in individual fecal samples of Koreans. (DOCX) [file pone.0022109.s004.docx]

Figure S5


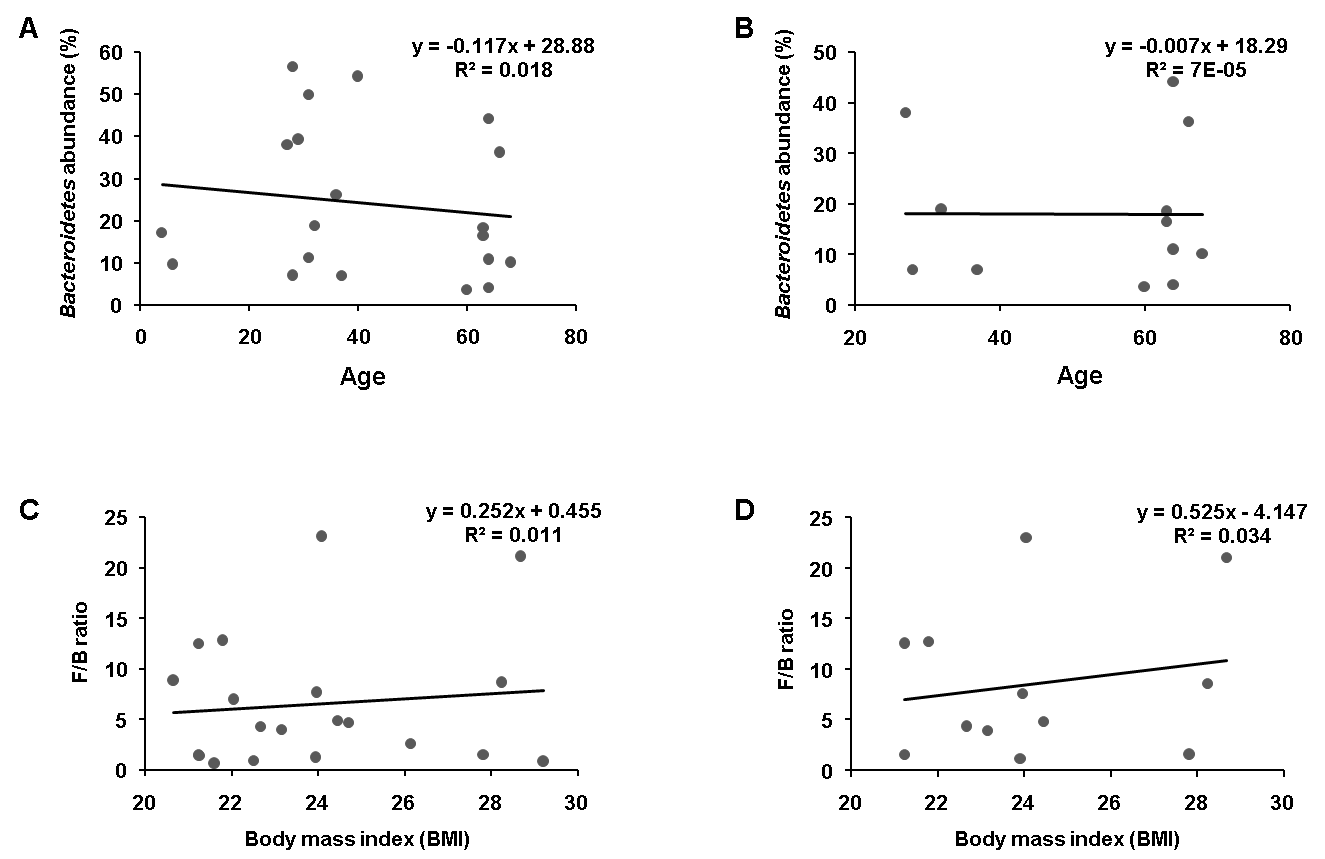

Supplement: Figure S5 — Correlation between age or BMI and bacterial estimates by pyrosequencing. The correlation between age and the relative abundance of Bacteroidetes in all individuals is shown in (A) and the combined data excluding 12 individuals are shown in (B). The correlation between body mass index (BMI) and the ratio of Firmicutes to Bacteroidetes in all individuals is shown in (C), whilst the combined data excluding 12 individuals are shown in (D). Bacterial abundances were determined by pyrosequencing of the V1/V2 region of the 16S rRNA gene from 20 Koreans. (DOCX) [file pone.0022109.s005.docx]

Figure S6


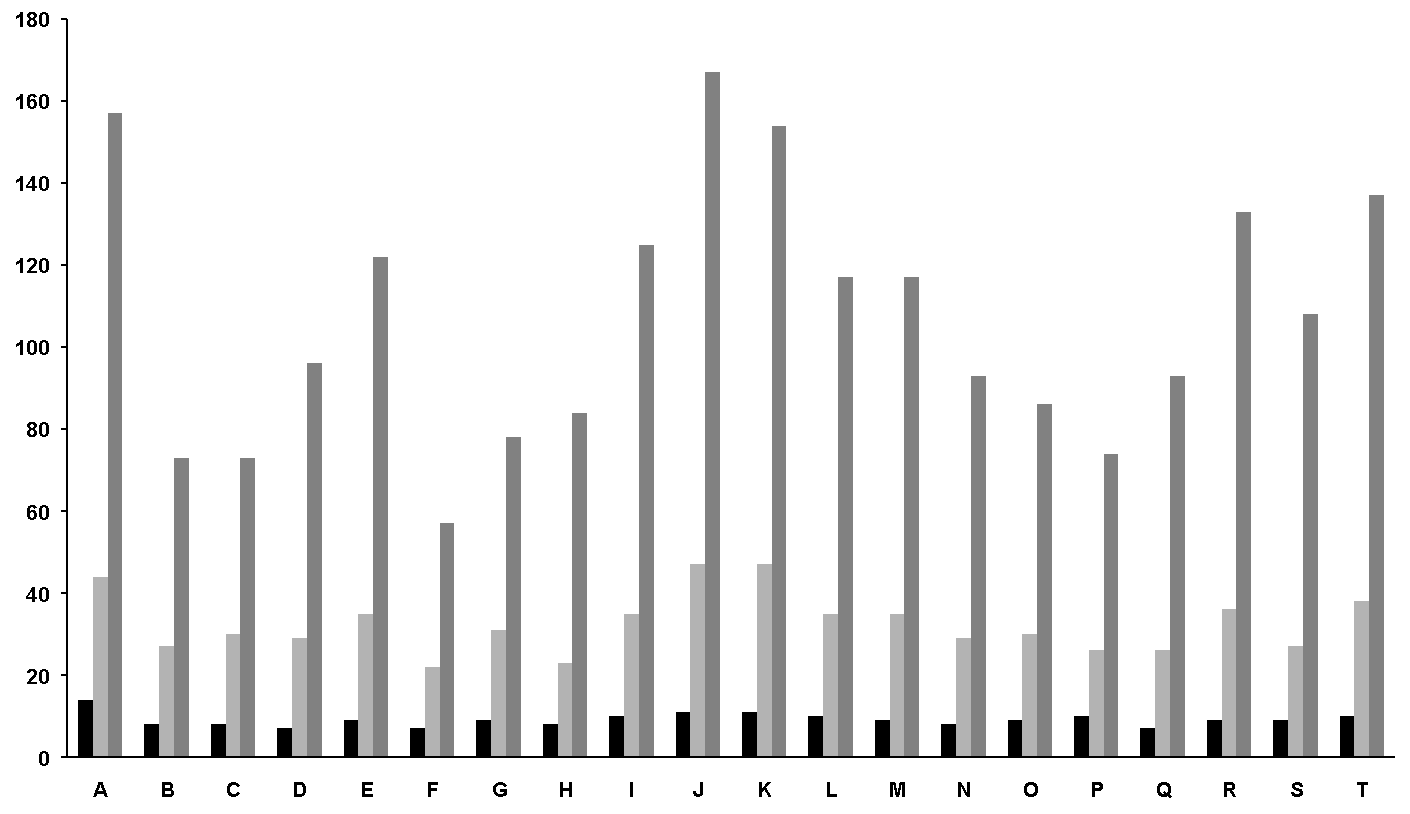

Supplement: Figure S6 — Distribution of taxa in each individual at different taxonomic levels. Black bars represent the number of phylum level taxa detected in each individual. The grey and dark grey bars represent family level and genus level taxa, respectively. (DOCX) [file pone.0022109.s006.docx]

Figure S7


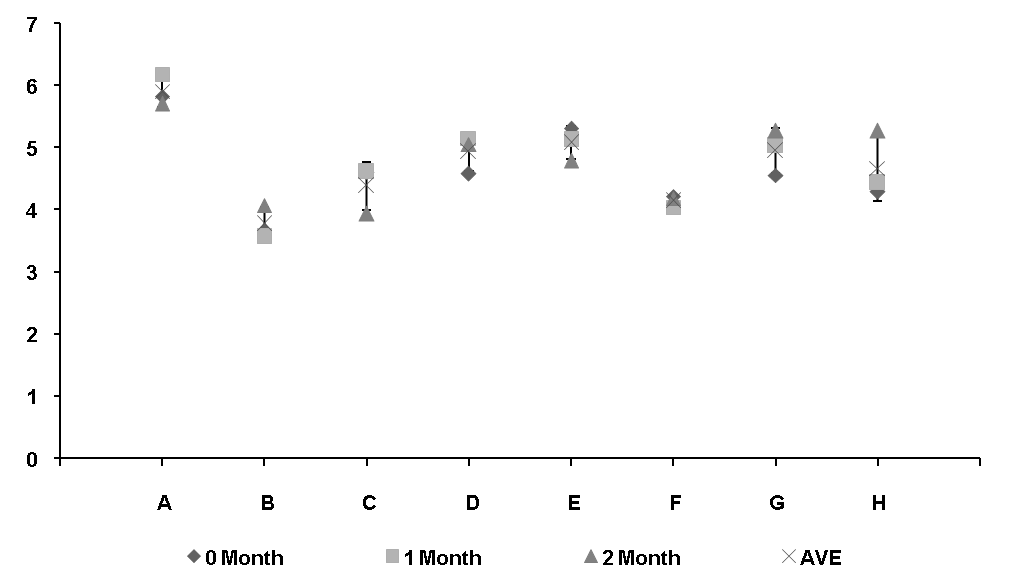

Supplement: Figure S7 — Variation in Shannon diversity index. Diversity indices of three samples from each of eight individuals are shown together with the average index of the three samples with SD. (DOCX) [file pone.0022109.s007.docx]
