# Supplementary material for: Comparative Analysis of Korean Human Gut Microbiota by Barcoded Pyrosequencing
Source: PLoS One. 2011 Jul 29;6(7):e22109. doi: 10.1371/journal.pone.0022109 (PMC3146482; doi:10.1371/journal.pone.0022109)
Supplement: Table S1 — The number of operational taxonomic units (OTUs) in each sample determined with various percentage identity thresholds. (DOCX) [file pone.0022109.s009.docx]

Table S1

| Sample ID | Read | Similarity cut off value (%) | | | | | | | | | | | |
| --- | --- | --- | --- | --- | --- | --- | --- | --- | --- | --- | --- | --- | --- |
|  |  | unique | 100 | 99 | 98 | 97 | 96 | 95 | 94 | 93 | 92 | 91 | 90 |
| A0 | 8505 | 3037 | 2787 | 1983 | 1498 | 1179 | 938 | 805 | 704 | 625 | 562 | 514 | 481 |
| A1 | 12006 | 4073 | 3678 | 2597 | 1904 | 1508 | 1193 | 1017 | 895 | 810 | 736 | 679 | 638 |
| A2 | 8143 | 2654 | 2437 | 1790 | 1353 | 1048 | 849 | 720 | 631 | 568 | 513 | 473 | 442 |
| B0 | 15411 | 2421 | 2208 | 1331 | 891 | 652 | 486 | 391 | 331 | 287 | 259 | 233 | 211 |
| B1 | 9757 | 1834 | 1643 | 1007 | 654 | 466 | 349 | 280 | 232 | 198 | 176 | 161 | 142 |
| B2 | 5103 | 1322 | 1195 | 778 | 549 | 408 | 309 | 245 | 219 | 191 | 169 | 156 | 142 |
| C0 | 7651 | 1934 | 1770 | 1162 | 792 | 590 | 434 | 356 | 303 | 261 | 237 | 219 | 200 |
| C1 | 13340 | 3006 | 2624 | 1500 | 986 | 702 | 508 | 401 | 324 | 276 | 243 | 217 | 203 |
| C2 | 9937 | 1921 | 1754 | 1095 | 764 | 570 | 431 | 362 | 309 | 271 | 244 | 226 | 207 |
| D0 | 7052 | 1897 | 1753 | 1164 | 811 | 626 | 502 | 407 | 352 | 310 | 282 | 263 | 251 |
| D1 | 8025 | 2388 | 2179 | 1450 | 1015 | 756 | 595 | 484 | 411 | 359 | 321 | 292 | 275 |
| D2 | 8090 | 2394 | 2180 | 1449 | 1002 | 741 | 583 | 483 | 418 | 365 | 327 | 304 | 285 |
| E0 | 4917 | 1756 | 1622 | 1206 | 883 | 673 | 542 | 454 | 395 | 359 | 325 | 301 | 285 |
| E1 | 12899 | 3563 | 3194 | 2041 | 1382 | 1001 | 786 | 635 | 552 | 488 | 438 | 398 | 376 |
| E2 | 10357 | 2900 | 2590 | 1680 | 1149 | 829 | 648 | 530 | 457 | 411 | 366 | 337 | 322 |
| F0 | 8340 | 1982 | 1761 | 1114 | 743 | 540 | 420 | 331 | 273 | 234 | 197 | 178 | 170 |
| F1 | 6110 | 1509 | 1400 | 937 | 648 | 488 | 387 | 318 | 274 | 230 | 206 | 183 | 177 |
| F2 | 5389 | 1421 | 1314 | 875 | 608 | 445 | 350 | 290 | 244 | 213 | 187 | 171 | 165 |
| G0 | 5709 | 1763 | 1528 | 949 | 652 | 468 | 354 | 292 | 257 | 222 | 205 | 190 | 177 |
| G1 | 3835 | 1438 | 1269 | 882 | 648 | 492 | 380 | 307 | 275 | 248 | 221 | 204 | 194 |
| G2 | 5538 | 1955 | 1738 | 1160 | 818 | 624 | 492 | 412 | 367 | 319 | 285 | 254 | 238 |
| H0 | 3385 | 1078 | 984 | 680 | 458 | 338 | 249 | 210 | 179 | 161 | 147 | 139 | 131 |
| H1 | 3683 | 1269 | 1142 | 739 | 492 | 357 | 260 | 209 | 184 | 158 | 140 | 122 | 116 |
| H2 | 13824 | 3610 | 3147 | 1942 | 1297 | 943 | 691 | 538 | 456 | 386 | 348 | 318 | 293 |
| I | 7075 | 2603 | 2349 | 1681 | 1253 | 948 | 747 | 620 | 539 | 478 | 434 | 399 | 371 |
| J | 10630 | 3735 | 3359 | 2371 | 1739 | 1356 | 1109 | 944 | 839 | 747 | 680 | 630 | 587 |
| K | 10679 | 3680 | 3259 | 2259 | 1657 | 1280 | 1010 | 837 | 751 | 668 | 601 | 545 | 503 |
| L | 3974 | 1720 | 1590 | 1234 | 976 | 778 | 642 | 561 | 506 | 459 | 416 | 382 | 358 |
| M | 9682 | 2893 | 2628 | 1854 | 1353 | 1030 | 814 | 686 | 604 | 526 | 465 | 426 | 394 |
| N | 6380 | 2247 | 2001 | 1321 | 950 | 725 | 553 | 464 | 405 | 362 | 322 | 288 | 261 |
| O | 5846 | 1819 | 1658 | 1168 | 864 | 667 | 530 | 440 | 379 | 327 | 291 | 267 | 248 |
| P | 2108 | 952 | 854 | 609 | 471 | 387 | 324 | 284 | 255 | 229 | 221 | 204 | 198 |
| Q | 7971 | 2770 | 2482 | 1686 | 1211 | 903 | 686 | 552 | 463 | 400 | 342 | 304 | 280 |
| R | 19781 | 5149 | 4509 | 2755 | 1869 | 1358 | 1038 | 840 | 711 | 615 | 541 | 471 | 433 |
| S | 4634 | 1767 | 1584 | 1122 | 850 | 684 | 565 | 494 | 429 | 380 | 341 | 315 | 292 |
| T | 13493 | 3876 | 3486 | 2310 | 1635 | 1212 | 931 | 760 | 660 | 573 | 505 | 462 | 425 |
| Average | 8313 | 2398 | 2157 | 1441 | 1023 | 771 | 602 | 499 | 433 | 381 | 341 | 312 | 291 |
| SD | 3858 | 966 | 851 | 561 | 406 | 313 | 251 | 212 | 188 | 169 | 153 | 140 | 130 |
