# Supplementary material for: Comparative Analysis of Korean Human Gut Microbiota by Barcoded Pyrosequencing
Source: PLoS One. 2011 Jul 29;6(7):e22109. doi: 10.1371/journal.pone.0022109 (PMC3146482; doi:10.1371/journal.pone.0022109)
Supplement: Table S2 — Relative abundance (%) of 38 genera from each individual. (DOCX) [file pone.0022109.s010.docx]

Table S2

| **Genus** | **A** | **B** | **C** | **D** | **E** | **F** | **G** | **H** | **I** | **J** | **K** | **L** | **M** | **N** | **O** | **P** | **Q** | **R** | **S** | **T** |
| --- | --- | --- | --- | --- | --- | --- | --- | --- | --- | --- | --- | --- | --- | --- | --- | --- | --- | --- | --- | --- |
| ***Faecalibacterium*** | **7.4** | **0.3** | **2.0** | **13.6** | **25.7** | **9.2** | **1.0** | **10.2** | **21.1** | **19.4** | **6.9** | **11.4** | **4.5** | **10.6** | **11.5** | **19.4** | **19.9** | **6.6** | **24.9** | **9.8** |
| ***Prevotella*** | **15.2** | **29.1** | **33.9** | **0.0** | **3.1** | **0.0** | **0.0** | **0.0** | **0.8** | **1.6** | **8.8** | **8.6** | **30.9** | **1.3** | **20.3** | **0.0** | **0.0** | **2.2** | **4.6** | **28.8** |
| ***Bacteroides*** | **5.0** | **9.6** | **11.1** | **33.7** | **3.7** | **51.3** | **5.6** | **7.6** | **2.0** | **2.1** | **2.0** | **2.7** | **0.7** | **2.5** | **15.9** | **2.0** | **8.5** | **8.4** | **1.6** | **1.7** |
| ***Clostridium*** | **7.5** | **3.3** | **7.4** | **4.7** | **5.4** | **8.6** | **23.1** | **6.6** | **6.0** | **8.4** | **9.3** | **10.6** | **7.0** | **13.9** | **5.3** | **8.9** | **11.0** | **8.4** | **9.5** | **8.0** |
| ***Ruminococcus*** | **7.2** | **5.2** | **6.4** | **10.0** | **1.5** | **4.0** | **18.6** | **5.3** | **1.4** | **5.4** | **0.7** | **1.4** | **1.3** | **4.0** | **10.7** | **1.8** | **5.1** | **19.0** | **2.7** | **1.8** |
| ***Eubacterium*** | **2.7** | **1.8** | **0.4** | **4.0** | **4.6** | **1.5** | **1.2** | **7.9** | **2.4** | **5.5** | **3.5** | **2.0** | **3.0** | **2.3** | **9.4** | **7.0** | **6.7** | **5.4** | **2.7** | **7.9** |
| ***Dialister*** | **1.8** | **0.0** | **0.1** | **0.0** | **15.8** | **0.0** | **10.9** | **0.0** | **23.6** | **6.4** | **1.0** | **8.2** | **0.0** | **0.1** | **0.1** | **0.3** | **1.7** | **6.4** | **1.1** | **3.4** |
| ***Oscillibacter*** | **7.0** | **0.0** | **0.7** | **0.9** | **3.7** | **0.0** | **0.2** | **1.0** | **4.7** | **3.9** | **17.0** | **9.1** | **3.6** | **2.0** | **0.7** | **0.4** | **0.8** | **0.4** | **7.1** | **3.7** |
| ***Roseburia*** | **0.9** | **0.1** | **0.1** | **1.2** | **1.7** | **1.1** | **2.9** | **1.5** | **0.7** | **1.5** | **4.5** | **5.5** | **1.1** | **5.9** | **0.1** | **13.2** | **2.2** | **0.2** | **0.6** | **2.0** |
| ***Fusobacterium*** | **2.3** | **2.3** | **1.7** | **0.0** | **8.2** | **0.5** | **0.6** | **1.2** | **1.0** | **2.7** | **4.5** | **0.9** | **6.5** | **0.8** | **1.5** | **4.8** | **0.3** | **4.4** | **1.8** | **0.5** |
| ***Lachnospira*** | **1.6** | **0.3** | **2.1** | **1.3** | **0.5** | **2.5** | **0.9** | **1.7** | **0.4** | **0.5** | **1.4** | **5.3** | **1.1** | **13.5** | **0.3** | **0.4** | **7.7** | **0.2** | **0.2** | **3.4** |
| ***Megamonas*** | **3.0** | **10.4** | **0.0** | **0.0** | **0.0** | **0.0** | **0.0** | **16.6** | **0.0** | **0.3** | **0.8** | **0.0** | **2.1** | **2.4** | **3.0** | **0.0** | **0.2** | **1.4** | **0.4** | **0.0** |
| ***Parabacteroides*** | **0.6** | **0.8** | **2.9** | **0.8** | **2.1** | **2.5** | **0.0** | **4.7** | **1.7** | **0.6** | **2.3** | **1.6** | **0.7** | **0.2** | **0.7** | **0.0** | **1.6** | **1.7** | **0.2** | **1.0** |
| ***Hydrogenoanaerobacterium*** | **0.1** | **0.0** | **0.0** | **0.0** | **0.0** | **0.0** | **0.0** | **1.2** | **4.3** | **2.6** | **1.5** | **0.6** | **5.7** | **0.0** | **0.5** | **4.0** | **0.0** | **4.1** | **0.1** | **0.0** |
| ***Subdoligranulum*** | **1.3** | **0.3** | **0.0** | **0.9** | **0.2** | **1.0** | **0.7** | **4.3** | **1.3** | **1.4** | **0.4** | **1.7** | **0.3** | **1.0** | **0.0** | **0.0** | **2.6** | **0.2** | **2.4** | **0.5** |
| ***Blautia*** | **0.3** | **0.8** | **6.2** | **0.1** | **0.1** | **0.2** | **1.8** | **0.0** | **0.0** | **0.0** | **0.1** | **0.4** | **0.0** | **0.3** | **0.1** | **3.7** | **0.6** | **1.0** | **0.0** | **0.0** |
| ***Alistipes*** | **1.2** | **0.0** | **0.9** | **0.9** | **0.9** | **0.0** | **0.0** | **3.6** | **1.8** | **1.0** | **1.1** | **0.5** | **0.2** | **0.0** | **0.8** | **0.4** | **0.0** | **0.2** | **0.0** | **0.1** |
| ***Coprococcus*** | **0.4** | **0.1** | **0.0** | **0.0** | **0.0** | **0.0** | **0.1** | **0.1** | **1.4** | **0.3** | **0.4** | **0.0** | **4.0** | **0.2** | **0.7** | **0.1** | **0.7** | **0.0** | **2.7** | **1.4** |
| ***Sutterella*** | **2.5** | **1.8** | **3.2** | **0.0** | **0.0** | **0.0** | **0.0** | **1.0** | **0.4** | **0.1** | **0.0** | **0.0** | **0.9** | **0.1** | **0.1** | **0.0** | **0.0** | **0.0** | **0.0** | **0.2** |
| ***Phascolarctobacterium*** | **0.1** | **0.0** | **0.0** | **1.0** | **0.0** | **1.7** | **0.0** | **0.0** | **0.0** | **0.0** | **0.0** | **0.0** | **0.0** | **0.0** | **0.7** | **5.2** | **0.0** | **0.0** | **0.0** | **0.3** |
| ***Spiroplasma*** | **0.2** | **0.0** | **0.0** | **0.0** | **3.0** | **0.0** | **0.0** | **0.0** | **0.7** | **2.5** | **1.0** | **0.1** | **0.0** | **0.0** | **0.0** | **0.0** | **0.0** | **0.0** | **0.6** | **0.5** |
| ***Veillonella*** | **0.1** | **0.0** | **0.2** | **0.0** | **0.0** | **0.0** | **0.4** | **0.6** | **0.4** | **0.0** | **0.0** | **0.0** | **0.0** | **2.4** | **0.0** | **2.2** | **0.3** | **0.0** | **0.2** | **0.1** |
| ***Klebsiella*** | **0.0** | **0.0** | **3.6** | **0.0** | **0.0** | **0.0** | **0.1** | **0.0** | **0.8** | **0.0** | **0.1** | **0.5** | **0.0** | **0.3** | **0.0** | **1.4** | **0.0** | **0.0** | **0.0** | **0.1** |
| ***Sporobacter*** | **0.0** | **0.0** | **0.0** | **0.0** | **0.0** | **0.0** | **0.0** | **0.8** | **3.6** | **0.1** | **1.6** | **0.4** | **0.0** | **0.1** | **0.0** | **0.0** | **0.0** | **0.0** | **0.0** | **0.0** |
| ***Oribacterium*** | **0.1** | **0.0** | **0.0** | **0.0** | **0.0** | **0.0** | **0.0** | **0.0** | **0.0** | **1.0** | **1.7** | **3.0** | **0.0** | **0.5** | **0.0** | **0.0** | **0.0** | **0.0** | **0.0** | **0.0** |
| ***Parasutterella*** | **0.0** | **0.0** | **0.0** | **1.8** | **0.0** | **0.3** | **0.0** | **0.0** | **0.0** | **0.0** | **0.1** | **0.0** | **0.0** | **0.0** | **1.6** | **0.0** | **1.4** | **0.0** | **0.4** | **0.1** |
| ***Shuttleworthia*** | **0.1** | **0.0** | **0.0** | **0.0** | **0.0** | **0.0** | **0.0** | **0.0** | **0.0** | **0.0** | **0.0** | **0.0** | **0.0** | **0.0** | **0.0** | **5.0** | **0.0** | **0.0** | **0.0** | **0.0** |
| ***Peptococcus*** | **0.3** | **0.0** | **0.0** | **0.0** | **0.0** | **0.0** | **0.0** | **0.0** | **0.0** | **0.3** | **0.1** | **0.2** | **0.0** | **0.1** | **0.0** | **0.0** | **0.0** | **0.0** | **3.4** | **0.0** |
| ***Paraprevotella*** | **0.2** | **0.3** | **0.0** | **0.0** | **0.4** | **0.0** | **0.0** | **0.0** | **0.1** | **0.8** | **0.1** | **0.1** | **0.1** | **0.0** | **1.4** | **0.0** | **0.0** | **0.1** | **0.2** | **0.2** |
| ***Escherichia*** | **0.0** | **0.0** | **0.0** | **0.2** | **0.0** | **0.3** | **2.8** | **0.0** | **0.3** | **0.0** | **0.0** | **0.0** | **0.0** | **0.0** | **0.0** | **0.0** | **0.0** | **0.0** | **0.0** | **0.1** |
| ***Streptococcus*** | **0.1** | **0.1** | **0.1** | **0.1** | **0.0** | **0.1** | **0.2** | **0.0** | **0.0** | **0.4** | **0.0** | **0.0** | **0.1** | **0.0** | **0.6** | **0.0** | **0.1** | **0.2** | **1.5** | **0.0** |
| ***Butyrivibrio*** | **0.6** | **0.0** | **0.0** | **0.0** | **0.1** | **0.0** | **0.0** | **0.0** | **0.0** | **0.6** | **0.1** | **0.2** | **0.0** | **0.1** | **0.0** | **1.1** | **0.0** | **0.0** | **0.0** | **0.0** |
| ***Succinispira*** | **0.0** | **0.8** | **1.9** | **0.0** | **0.0** | **0.0** | **0.0** | **0.0** | **0.0** | **0.0** | **0.0** | **0.0** | **0.1** | **0.0** | **0.0** | **0.0** | **0.0** | **0.0** | **0.0** | **0.0** |
| ***Barnesiella*** | **0.3** | **0.0** | **0.0** | **0.1** | **0.6** | **0.0** | **0.0** | **0.0** | **0.0** | **1.3** | **0.1** | **0.0** | **0.0** | **0.0** | **0.0** | **0.3** | **0.0** | **0.0** | **0.0** | **0.0** |
| ***Turicibacter*** | **0.0** | **0.0** | **0.1** | **0.0** | **0.0** | **0.6** | **0.0** | **0.0** | **0.0** | **0.1** | **0.0** | **0.6** | **0.0** | **0.1** | **0.0** | **0.9** | **0.0** | **0.0** | **0.0** | **0.0** |
| ***Syntrophomonas*** | **0.0** | **0.0** | **0.0** | **0.0** | **0.1** | **0.0** | **0.0** | **0.0** | **0.1** | **0.2** | **0.0** | **0.1** | **0.1** | **0.0** | **0.0** | **0.0** | **0.7** | **0.0** | **0.0** | **0.0** |
| ***Bacteriovorax*** | **0.1** | **0.0** | **0.0** | **0.0** | **0.0** | **0.0** | **0.0** | **0.0** | **0.0** | **0.0** | **0.0** | **0.5** | **0.0** | **0.0** | **0.0** | **0.0** | **0.0** | **0.0** | **0.7** | **0.2** |
| ***Oscillospira*** | **0.0** | **0.0** | **0.0** | **0.0** | **0.0** | **0.0** | **0.0** | **0.0** | **0.7** | **0.0** | **0.0** | **0.0** | **0.0** | **0.0** | **0.0** | **0.0** | **0.0** | **0.0** | **0.0** | **0.0** |
