# Supplementary material for: Comparative Analysis of Korean Human Gut Microbiota by Barcoded Pyrosequencing
Source: PLoS One. 2011 Jul 29;6(7):e22109. doi: 10.1371/journal.pone.0022109 (PMC3146482; doi:10.1371/journal.pone.0022109)
Supplement: Table S3 — List of gut microbiota detected with more than 1% abundance in members of four countries. (DOCX) [file pone.0022109.s011.docx]

Table S3

| Phylum | Taxon name | Korea | China | US | Japan |
| --- | --- | --- | --- | --- | --- |
| Acidobacteria | *Bifidobacterium adolescentis* | 0.0% | 0.8% | 0.2% | 4.4% |
| Acidobacteria | *Bifidobacterium bifidum* | 0.0% | 0.0% | 0.1% | 1.5% |
| Acidobacteria | *Bifidobacterium longum* | 0.0% | 0.7% | 0.1% | 10.3% |
| Acidobacteria | *Bifidobacterium* sp. Eg1 | 0.0% | 0.0% | 0.0% | 1.5% |
| Acidobacteria | *Bifidobacterium thermacidophilum* | 0.0% | 0.3% | 0.2% | 2.9% |
| Acidobacteria | *Collinsella aerofaciens* | 0.1% | 0.1% | 1.3% | 0.0% |
| Acidobacteria | *Collinsella* sp. CB20 | 0.0% | 0.0% | 0.0% | 1.5% |
| Bacteroidetes | *Bacteroides coprocola* | 2.3% | 0.0% | 0.8% | 0.0% |
| Bacteroidetes | *Bacteroides eggerthii* | 1.8% | 0.0% | 0.1% | 0.0% |
| Bacteroidetes | *Bacteroides fragilis* | 0.3% | 3.1% | 0.5% | 0.0% |
| Bacteroidetes | *Bacteroides galacturonicus* | 1.0% | 2.7% | 1.1% | 1.5% |
| Bacteroidetes | *Bacteroides ovatus* | 0.5% | 4.4% | 0.8% | 0.0% |
| Bacteroidetes | *Bacteroides* sp. CB57 | 0.4% | 8.0% | 1.8% | 0.0% |
| Bacteroidetes | *Bacteroides* sp. CJ89 | 0.2% | 2.1% | 0.3% | 1.5% |
| Bacteroidetes | *Bacteroides* sp. D8 | 1.7% | 6.5% | 0.7% | 2.9% |
| Bacteroidetes | *Bacteroides uniformis* | 0.6% | 2.9% | 1.4% | 1.5% |
| Bacteroidetes | *Bacteroides vulgatus* | 4.0% | 0.4% | 3.5% | 0.0% |
| Bacteroidetes | *Odoribacter splanchnicus* | 0.3% | 0.0% | 0.1% | 1.5% |
| Bacteroidetes | *Prevotella copri* | 3.4% | 0.0% | 0.3% | 0.0% |
| Bacteroidetes | *Prevotella* sp. BI-42 | 5.0% | 0.0% | 0.2% | 0.0% |
| Bacteroidetes | *Prevotella* sp. DJF RP53 | 1.0% | 0.0% | 0.0% | 0.0% |
| Bacteroidetes | *Prevotella stercorea* | 1.1% | 0.0% | 0.0% | 0.0% |
| Bacteroidetes | Bacteroidetes enrichment culture LET-13 | 0.0% | 0.0% | 0.0% | 1.5% |
| Firmicutes | *Howardella ureilytica* | 0.1% | 0.1% | 0.2% | 1.5% |
| Firmicutes | Butyrate-producing bacterium A2-207 | 0.6% | 0.2% | 0.3% | 1.5% |
| Firmicutes | Butyrate-producing bacterium A2-232 | 0.1% | 0.0% | 0.3% | 1.5% |
| Firmicutes | Butyrate-producing bacterium M21/2 | 1.8% | 1.1% | 1.3% | 2.9% |
| Firmicutes | Clostridiaceae bacterium FH052 | 0.3% | 0.4% | 1.0% | 0.0% |
| Firmicutes | *Clostridium lituseburense* | 0.2% | 0.0% | 0.3% | 1.5% |
| Firmicutes | *Clostridium methoxybenzovorans* | 0.1% | 0.0% | 0.1% | 1.5% |
| Firmicutes | *Clostridium saccharobutylicum* | 0.0% | 0.0% | 0.0% | 1.5% |
| Firmicutes | *Clostridium* sp. CM-C99 | 0.0% | 0.0% | 0.0% | 1.5% |
| Firmicutes | *Clostridium* sp. SC106 | 0.8% | 0.3% | 0.7% | 1.5% |
| Firmicutes | *Clostridium symbiosum* | 0.9% | 0.0% | 1.5% | 0.0% |
| Firmicutes | *Clostridium cocleatum* | 0.6% | 0.3% | 1.4% | 1.5% |
| Firmicutes | *Clostridium innocuum* | 0.0% | 0.1% | 0.0% | 1.5% |
| Firmicutes | *Clostridium rectum* | 1.9% | 0.0% | 0.0% | 0.0% |
| Firmicutes | *Carboxydocella ferrireducens* | 0.2% | 1.2% | 0.3% | 1.5% |
| Firmicutes | *Eubacterium eligens* | 0.5% | 0.7% | 0.6% | 4.4% |
| Firmicutes | *Eubacterium rectale* | 1.2% | 8.8% | 4.4% | 1.5% |
| Firmicutes | *Anaerostipes* sp. 35-7 | 0.1% | 0.0% | 0.1% | 1.5% |
| Firmicutes | *Coprococcus catus* | 0.1% | 0.0% | 0.1% | 1.5% |
| Firmicutes | *Coprococcus eutactus* | 0.5% | 2.1% | 0.2% | 0.0% |
| Firmicutes | *Lachnospira pectinoschiza* | 1.3% | 0.7% | 0.9% | 1.5% |
| Firmicutes | *Roseburia intestinalis* | 0.8% | 1.1% | 0.7% | 0.0% |
| Firmicutes | *Roseburia inulinivorans* | 0.5% | 2.4% | 0.8% | 0.0% |
| Firmicutes | *Oscillibacter valericigenes* | 0.4% | 0.3% | 0.6% | 1.5% |
| Firmicutes | *Faecalibacterium prausnitzii* | 9.5% | 7.3% | 8.5% | 4.4% |
| Firmicutes | *Faecalibacterium* sp. DJF VR20 | 2.1% | 1.0% | 0.9% | 1.5% |
| Firmicutes | *Ruminococcus albus* | 0.0% | 2.1% | 0.4% | 0.0% |
| Firmicutes | *Ruminococcus gnavus* | 1.5% | 0.1% | 0.3% | 0.0% |
| Firmicutes | *Ruminococcus* sp. CJ60 | 0.4% | 0.0% | 0.4% | 1.5% |
| Firmicutes | *Ruminococcus* sp. CO1 | 0.8% | 0.1% | 2.1% | 1.5% |
| Firmicutes | *Ruminococcus* sp. CO47 | 0.4% | 0.3% | 1.3% | 0.0% |
| Firmicutes | *Ruminococcus* sp. SC103 | 0.3% | 0.7% | 0.7% | 1.5% |
| Firmicutes | *Subdoligranulum* sp. DJF VR33k2 | 1.2% | 1.0% | 0.8% | 4.4% |
| Firmicutes | *Acidaminococcus* sp. DJF RP55 | 0.0% | 0.0% | 0.1% | 1.5% |
| Firmicutes | *Dialister invisus* | 0.5% | 1.1% | 0.4% | 0.0% |
| Firmicutes | *Dialister* sp. E2 20 | 1.5% | 3.7% | 1.0% | 0.0% |
| Firmicutes | *Dialister succinatiphilus* | 1.8% | 0.0% | 0.5% | 0.0% |
| Firmicutes | *Megamonas hypermegale* | 2.8% | 0.0% | 0.0% | 0.0% |
| Firmicutes | *Succinispira mobilis* | 0.3% | 0.0% | 0.1% | 1.5% |
| Firmicutes | *Catenibacterium mitsuokai* | 0.0% | 0.0% | 0.2% | 1.5% |
| Firmicutes | Uncultured firmicute bacterium ASF500 | 0.1% | 0.0% | 0.2% | 1.5% |
| Proteobacteria | *Sutterella wadsworthensis* | 0.1% | 1.3% | 0.1% | 0.0% |
| Proteobacteria | *Bilophila wadsworthia* | 0.0% | 0.0% | 0.0% | 1.5% |
| Proteobacteria | *Klebsiella pneumoniae* | 0.3% | 0.0% | 0.0% | 2.9% |
| Verrucomicrobia | *Akkermansia muciniphila* | 0.0% | 0.2% | 1.4% | 0.0% |
| Unclassified | Uncultured bacterium 10C29 | 0.3% | 0.1% | 0.4% | 1.5% |
| Unclassified | Mpn-isolate group 24 | 0.4% | 0.2% | 1.3% | 0.0% |
| Unclassified | Human intestinal bacterium PUE | 0.6% | 0.1% | 1.1% | 0.0% |
| Unclassified | Human intestinal firmicute CB17 | 0.4% | 0.3% | 0.6% | 2.9% |
| Unclassified | Human intestinal firmicute CO19 | 2.2% | 1.2% | 0.9% | 0.0% |
| Unclassified | Human intestinal firmicute CO4 | 0.2% | 0.1% | 0.6% | 1.5% |
| Unclassified | Swine fecal bacterium FPC70 | 0.7% | 0.2% | 2.6% | 4.4% |
| Unclassified | Unidentified thermophilic eubacterium ST10 | 0.1% | 0.0% | 0.1% | 1.5% |
