# Supplementary material for: Comparative Analysis of Korean Human Gut Microbiota by Barcoded Pyrosequencing
Source: PLoS One. 2011 Jul 29;6(7):e22109. doi: 10.1371/journal.pone.0022109 (PMC3146482; doi:10.1371/journal.pone.0022109)
Supplement: Table S4 — Primers used in this study and references for the amplification of human gut bacterial 16S rRNA genes. (DOCX) [file pone.0022109.s012.docx]

Table S4

| Country | Primers | Coverages | Studies |
| --- | --- | --- | --- |
| Korea | 8F-1D^a^: AGAGTTTGATCMTGGCTCAG | (213233/1542892) | This study |
|  | 518R-1D: WTTACCGCGGCTGCTGG | (1121998/1542892) |  |
|  |  |  |  |
| US | 8F: AGAGTTTGATCCTGGCTCAG | (172480/1542892) | [2] |
|  | 1391R-1D: GACGGGCGGTGTGTRCA | (395101/1542892) |  |
|  |  |  |  |
|  | 8F-1D^:^ AGAGTTTGATCMTGGCTCAG | (213233/1542892) | [33] |
|  | 1391R-1D: GACGGGCGGTGTGTRCA | (395101/1542892) |  |
|  |  |  |  |
|  | 8F: AGAGTTTGATCCTGGCTCAG | (172480/1542892) | [24] |
|  | 1391R-2D: GACGGGCGGTGWGTRCA | (425352/1542892) |  |
|  |  |  |  |
|  | 8F: AGAGTTTGATCCTGGCTCAG | (172480/1542892) | [35] |
|  | 1525R: AAGGAGGTGATCCAGCC | (26716/1542892) |  |
|  |  |  |  |
| China | 8F-1D^:^ AGAGTTTGATCMTGGCTCAG | (213233/1542892) | [33] |
|  | 1391R-1D: GACGGGCGGTGTGTRCA | (395101/1542892) |  |
|  |  |  |  |
| Japan | Metagenomic |  | [52] |

^a^D: the number of degeneracy
